# Transforming growth factor-β in tumour development

**DOI:** 10.3389/fmolb.2022.991612

**Published:** 2022-10-04

**Authors:** Charles B. Trelford, Lina Dagnino, Gianni M. Di Guglielmo

**Affiliations:** ^1^ Department of Physiology and Pharmacology, Schulich School of Medicine and Dentistry, Western University, London, ON, Canada; ^2^ Department of Oncology, Children’s Health Research Institute and Lawson Health Research Institute, London, ON, Canada

**Keywords:** transforming growth factor-b (TGFb), Smad, receptor trafficking, epithelial-mesenchymal transition (EMT), autophagy, protein 62/sequestosome 1 (p62/SQSTM1)

## Abstract

Transforming growth factor-β (TGFβ) is a ubiquitous cytokine essential for embryonic development and postnatal tissue homeostasis. TGFβ signalling regulates several biological processes including cell growth, proliferation, apoptosis, immune function, and tissue repair following injury. Aberrant TGFβ signalling has been implicated in tumour progression and metastasis. Tumour cells, in conjunction with their microenvironment, may augment tumourigenesis using TGFβ to induce epithelial-mesenchymal transition, angiogenesis, lymphangiogenesis, immune suppression, and autophagy. Therapies that target TGFβ synthesis, TGFβ-TGFβ receptor complexes or TGFβ receptor kinase activity have proven successful in tissue culture and in animal models, yet, due to limited understanding of TGFβ biology, the outcomes of clinical trials are poor. Here, we review TGFβ signalling pathways, the biology of TGFβ during tumourigenesis, and how protein quality control pathways contribute to the tumour-promoting outcomes of TGFβ signalling.

## Introduction

Transforming growth factor-β (TGFβ), a central modulator of development, growth, proliferation, immune function, apoptosis, and homeostasis, plays key roles in cellular communication (Hajek et al., 2012). TGFβ is secreted as a latent cytokine that is sequestered by extracellular matrix (ECM) proteins (Isogai et al., 2003). Following enzymatic or allosteric-mediated release and subsequent activation of TGFβ, TGFβ ligands bind to ubiquitously expressed cell surface receptors (Horiguchi et al., 2012). Autocrine or paracrine TGFβ signalling modulates cell function by regulating transcription, translation, and post-translational modifications of several proteins (Massagué, 2012). Alterations in TGFβ signalling pathways have been implicated in numerous pathologies, including congenital diseases, fibrotic disorders, immune dysfunction, and tumourigenesis (Massagué, 2008; Neuzillet et al., 2015). The regulation of TGFβ signalling in cancer is complex, as it generally plays a tumour suppressive role in normal tissues and early tumour development (Principe et al., 2014). In contrast, mutations or abnormalities in the tumour suppressive arms of TGFβ signalling are common in advanced cancers (Harradine and Akhurst, 2006). In tumour cells, this cytokine drives tumourigenesis by inducing epithelial-mesenchymal transition (EMT), metastasis, angiogenesis, autophagy, and immune supression (Bierie and Moses, 2006). In this review, we will discuss TGFβ signalling pathways and how TGFβ may progress tumourigenesis.

## Transforming growth factor-β pathways

The TGFβ superfamily consists of 33-members of secreted cytokines that are ubiquitously expressed in vertebrates and invertebrates. This superfamily includes TGFβ proteins, bone morphogenetic proteins (BMPs), activins, inhibins, nodal, lefty1, lefty2, anti-muellerian hormone (AMH), growth differentiation factors (GDFs), myostatin, and glial cell-derived neurotrophic factor (GDNF) (Lichtman et al., 2016). On the basis of their biological functions and mature protein structure, these members can be subclassified into four subfamilies (David and Massagué, 2018). In humans, the TGFβ subfamily consists of TGFβ1, TGFβ2, TGFβ3, the activin/inhibin/nodal subfamily consists of activinA, activinB, nodal, lefty1, lefty2, inhibinα, inhibinβ, the BMP/GDF subfamily consists of nine BMPs, and nine GDFs, and the fourth subfamily that has no defined relationship includes AMH, BMP15, GDF9, GDF15, and GDNF (Mueller and Nickel, 2012).

As homodimers or heterodimers, TGFβ superfamily members signal through heteromeric TGFβ receptor complexes. Seven different type I receptors, five type II receptors, and betaglycan and endoglin type III receptors have been described in vertebrates and invertebrates (Weiss and Attisano, 2013). Receptor activation leads to signalling cascades modulated by several classes of Sma-mothers against decapentaplegic (Smad) proteins, such as receptor regulated Smads (R-Smads), common Smads (co-Smads), and inhibitory Smads (I-Smads) (Massagué, 2012) as well as non-Smad signalling proteins (Mu et al., 2012). Although an extensive number of TGFβ superfamily members activate specific subsets of receptors and signalling molecules, this review will focus on the TGFβ subfamily.

### Synthesis and post-translational modifications of TGFβ

**FIGURE 1:**
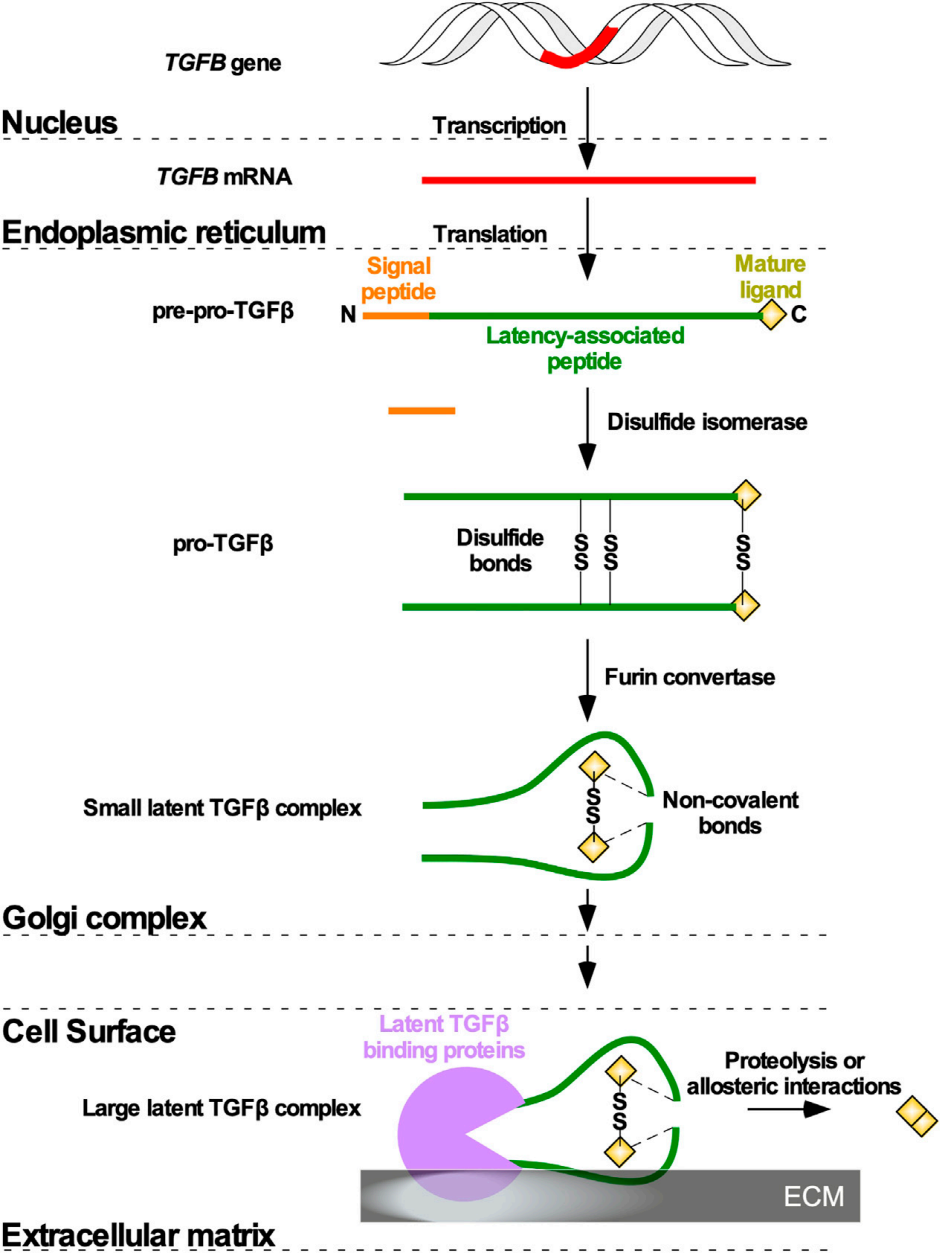
TGFβ ligand maturation. Following *Transforming Growth Factor-β* (*TGFB*) gene (red) transcription and *TGFB* mRNA translation in the nucleus and endoplasmic reticulum, respectively, TGFβ is synthesized as a precursor pro-TGFβ (pre-pro-TGFβ). Pre-pro-TGFβ contains an amino (N)-terminal signal peptide latency-associated peptide, and mature ligand. The N terminal signal peptide ensures transportation to the Golgi complex. In the Golgi complex, the signal peptide is cleaved, and disulfide isomerases catalyze disulfide bonds (SS) between two pre-pro-TGFβ monomers to generate pro-TGFβ. Furin convertases modify the latency-associated peptides, which non-covalently associate with mature ligands to generate a small latent TGFβ complex. The small latent TGFβ complex is secreted from the cell and attaches to latent TGFβ binding proteins in the extracellular matrix to form a large latent TGFβ complex. Mature ligands are released from the large latent TGFβ complexes *via* allosteric interactions or proteolysis mediated by enzymes.

In most metazoans, three genes encoding TGFβ isoforms have been described, and in humans the *TGFB1*, *TGFB2*, and *TGFB3* genes are located on chromosomes 19, 1, and 14, respectively (Nishimura et al., 1993; Cruts et al., 1995; Green et al., 2001). Although *TGFB1*, *TGFB2*, and *TGFB3* genes are highly conserved across species, there are some exceptions. For instance, *TGFB4* has been identified in avian species; however, genetic mapping of chicken *TGFB4* suggested that it is orthologous to human *TGFB1* (Halper et al., 2004)*.* Moreover, some South African frogs (*Xenopus laevis*) express a *tgfb5* gene (Kondaiah et al., 1990). Translation of the *TGFB1, TGFB2*, and *TGFB3* mRNA generates precursor polypeptides termed pre-pro-TGFβ, which are composed, respectively of 390, 412, and 412 amino acid residues (Khalil, 1999). The pre-pro-TGFβ species are composed of a signal peptide, a large amino-terminal latency-associated peptide (LAP), which ensures proper folding and transportation through the Golgi complex, and the residues of the mature ligand (Principe et al., 2014). Following signal peptide removal, disulfide isomerase catalyzes the formation of three disulfide bonds between two pre-pro-TGFβ monomers, linking cysteine residues at two positions in the LAP and one position in what will become the mature ligand. This modification gives rise to pro-TGFβ (Gentry et al., 1988). Within the Golgi complex membrane, furin and other convertases cleave LAP to generate small latent TGFβ complexes. Non-covalent bonds tether LAP to TGFβ, rendering the latter inactive (Poniatowski et al., 2015). Small latent TGFβ complexes, composed of a mature 25 kDa TGFβ dimer and two LAP moieties, are subsequently packaged into secretory vesicles in the Golgi complex (Dubois et al., 1995). Once secreted from the cell, the small latent TGFβ complexes are retained in the extracellular matrix (ECM), bound to latent TGFβ binding proteins (LTBPs) to form large latent TGFβ complexes (Massagué, 2012; Principe et al., 2014). TGFβ dimers can subsequently be released from the large latent TGFβ complexes through various enzymatic reactions or allosteric mechanisms (Figure 1) (Wipff et al., 2007; Tatti et al., 2008).

The enzymatic activation of TGFβ through proteolysis requires matrix metalloproteinases (MMPs), plasmin, and other proteases (Kobayashi et al., 2014; Korol et al., 2014). MMP2 and MMP9 are Ca^2+^-dependent Zn^+2^-containing endopeptidases that target the LAP-binding domains of LTBPs, releasing TGFβ from the large latent TGFβ complexes. Plasmin generated at the cell surface, following plasminogen cleavage by urokinase plasminogen, also contributes to TGFβ release from LAPs (Yee et al., 1993; Yu and Stamenkovic, 2000). Alternatively, allosteric activation of TGFβ is dependent on several LAP-binding cell surface proteins, such as thrombospondin-1, mannose 6-phosphate receptors, and integrins, which induce conformational rearrangements of LAP (Dennis and Rifkin, 1991; Schultz-Cherry and Murphy-Ullrich, 1993; Sarrazy et al., 2014; Takasaka et al., 2018). Modifications of LAP are also induced by reactive oxygen species (Pociask et al., 2004) as well as acidic (pH < 2) or basic (pH > 12) environments (Lyons et al., 1988). Since these diverse LAP conformers no longer favour binding to TGFβ, the latter is released from the large latent TGFβ complexes.

### Smad-dependent TGFβ signalling

After TGFβ ligands are released from large latent TGFβ complexes, they bind to cognate cell surface receptors. The Type I and II TGFβ receptors (TGFβRI and TGFβRII) exhibit serine-threonine kinase activity, and initiate signalling cascades upon ligand stimulation (Wrana et al., 1994). Type III TGFβ receptors (TGFβRIIIs) do not exhibit catalytic activity, but may facilitate the interaction between TGFβ ligands and TGFβRII (López-Casillas et al., 1994; Mclean and Di Guglielmo, 2010). TGFβ signalling is initiated when TGFβ binds to TGFβRII, triggering the association and phosphorylation of the glycine/serine domain of TGFβRI (Massagué, 2012). TGFβRI in turn phosphorylates downstream intracellular signalling molecules to induce canonical Smad-dependent and non-canonical Smad-independent TGFβ signalling, respectively (Massagué et al., 2000; Gunaratne and DiGuglielmo, 2013; McLean et al., 2013; Gunaratne et al., 2014).

**FIGURE 2:**
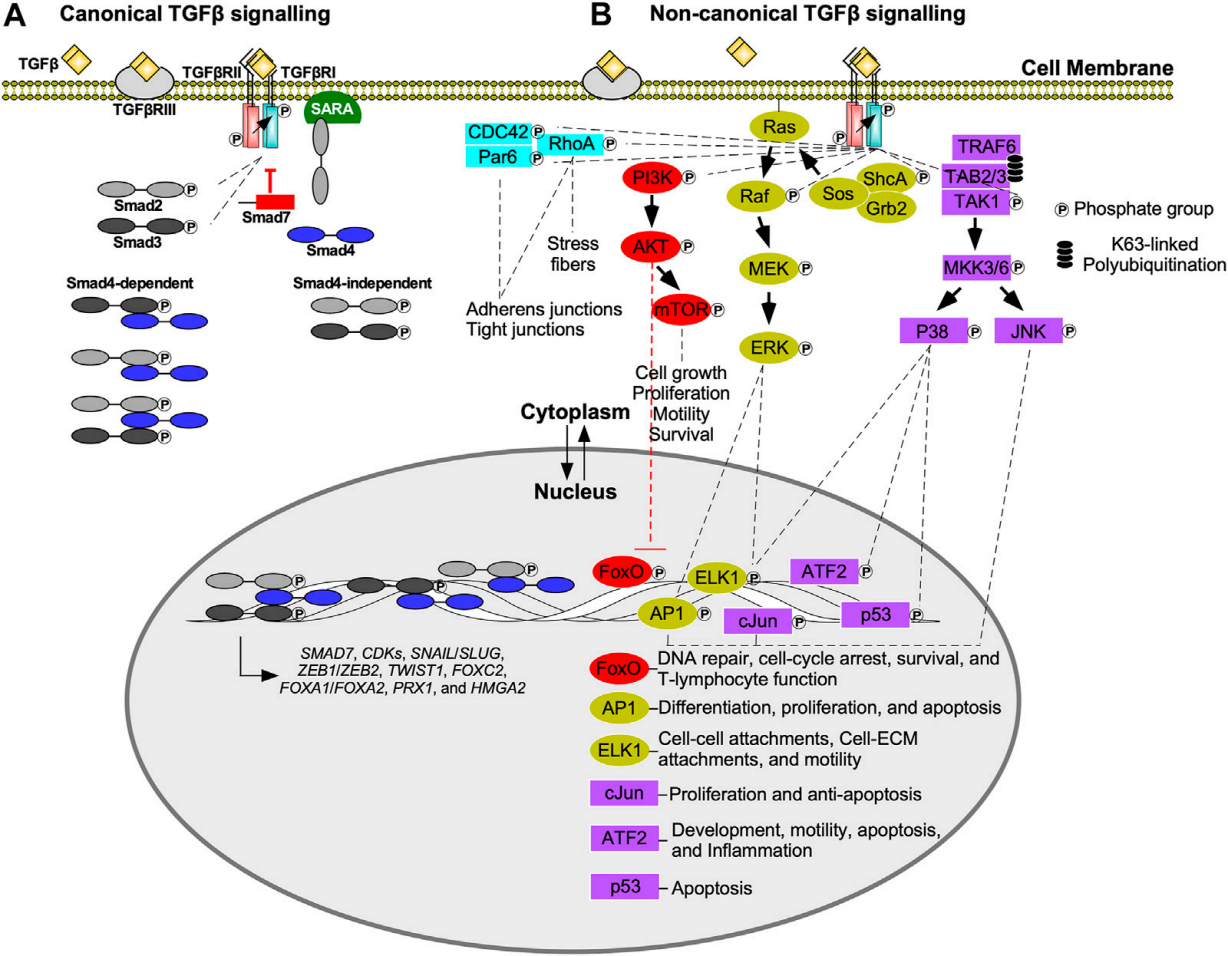
Canonical (Smad-dependent) and non-canonical (Smad-independent) TGFβ signalling. **(A)** Transforming growth factor-β (TGFβ) receptor type III (TGFβRIII) presents TGFβ to the type II receptor (TGFβRII). The TGFβ-TGFβRII complex phosphorylates TGFβ receptor type I (TGFβRI), which in turn phosphorylates R-Smads, Smad2 or Smad3. Phosphorylated Smad2/3 are released from the Smad anchor for receptor activation (SARA) protein, and translocate into the nucleus or form heterodimers/heterotrimers with Smad4 prior to nuclear translocation. Once in the nucleus, Smads function as transcription factors or interact with other transcription factors to regulate gene expression. Examples of genes regulated by Smads include, cyclin-dependent kinases (CDKs), Snail Family Transcriptional Repressor 1 and 2 (*SNAIL/SLUG*), Zinc Finger E-box Binding Homeobox one and 2 (*ZEB1/ZEB2*), Twist-related Protein 1 (*TWIST1*), Forkhead box C2 (*FOXC2*), Forkhead box A1 (*FOXA1*), Forkhead box A2 (*FOXA2*), Paired-related Homeobox 1 (*PRX1*), High Mobility Group AT-hook 2 (*HMGA2*), and *SMAD7*—which in turn dampens TGFβ signal transduction. **(B)** In non-canonical transforming growth factor-β (TGFβ) signalling, TGFβ receptor type I (TGFβRI) phosphorylates numerous downstream signalling molecules including TGFβ-activated kinase 1 (TAK1), src homology domain containing protein A (ShcA), and phosphoinositide 3-kinase (PI3K). Although partitioning defective six homolog (Par6) binds to TGFβRI, it is phosphorylated by TGFβRII. TGFβRI kinase activity is also important for Ras homolog family member A (RhoA) and cell division control protein 42 (CDC42) activation. The Par6/CDC42/RhoA pathway regulates adherens junctions, tight junctions, and stress fiber formation. PI3K phosphorylates protein kinase B (AKT), which inhibits Forkhead box O (FoxO) transcription factors that regulate genes responsible for DNA repair, cell cycle arrest, survival, and T-lymphocyte function. AKT also regulates cell growth, proliferation, motility, and survival by activating mechanistic target of rapamycin (mTOR). After ShcA is phosphorylated, it forms a complex with growth factor receptor bound 2 (Grb2) and sons of sevenless (Sos) to phosphorylate membrane bound Ras. This initiates a signalling cascade involving mitogen-activated protein kinase kinase (Raf), mitogen-activated protein kinase (MEK), and extracellular signal-regulated kinase 1 (ERK1). ERK1 upregulates activator protein 1 (AP1) and E-twenty-six Like-1 Protein (ELK1) transcription factors. AP1 upregulates genes that regulate differentiation, proliferation, and apoptosis, whereas ELK1 upregulates genes involved with cell-cell attachments, cell-extracellular matrix (ECM) attachments, and motility. TGFβRI phosphorylation promotes lysine (K)63-linked polyubiquitination of tumour necrosis factor receptor-associated factor 6 (TRAF6). TRAF6 forms a complex with TAK1 binding protein two and 3 (TAB2 and TAB3) to recruit TAK1. TGFβRI phosphorylates TAK1, which initiates signalling cascades that phosphorylate mitogen-activated protein kinase 3/6 (MKK3/6). MKK3/6 phosphorylates c-Jun amino-terminal kinase (JNK) and p38 MAPK. JNK regulates c-Jun and AP1 transcription factors, whereas p38 MAPK regulates activating transcription factor 2 (ATF2), p53, and ELK1 transcription factors. cJun upregulates genes involved with proliferation and survival, whereas ATF2 upregulates genes that modulate development, motility, apoptosis, and inflammation.

All three classes of Smad proteins, R-Smads (Smad2/3), co-Smad (Smad4), and I-Smads (Smad6/7), temporally regulate TGFβ signalling (Massagué et al., 2005). Signal initiation begins when TGFβRI phosphorylates Smad2 or Smad3 on the carboxyl (C) terminus serine-serine-x-serine (SSXS) motif. Phosphorylated Smad2/3 is then released from the Smad anchor for receptor activation (SARA) protein into the cytoplasm (Tsukazaki et al., 1998; Qin et al., 2002), where it can form hetero-dimeric or hetero-trimeric complexes with Smad4 (Massague and Wotton, 2000; David and Massagué, 2018). These complexes subsequently translocate into the nucleus, where they regulate gene expression directly, by activating transcription, or indirectly by modulating the activity of other transcription factors (Finnson et al., 2013). Smad targeted genes include I-Smads (Chen et al., 1999), cyclin-dependent kinase 4 (CDK4) (Ewen et al., 1995), and EMT-transcription factors, including Snail Family Transcriptional Repressor one and 2 (SNAIL and SLUG), Zinc Finger E-box Binding Homeobox 1 and 2 (ZEB1 and ZEB2), Twist-related Protein 1 (TWIST1), Forkhead box C2 (FOXC2), Forkhead box A1 (FOXA1), Forkhead box A2 (FOXA2), Paired-related Homeobox 1 (PRX1), and High Mobility Group AT-hook 2 (HMGA2; Figure 2A) (Hajek et al., 2012; Katsuno et al., 2013). Through negative feedback mechanisms, Smad6 and Smad7 terminate TGFβ pathway activation (Figure 2A). I-Smads block R-Smad access to TGFβRI or recruit phosphatases (Iyengar, 2017; Kim and Baek, 2018), leading to dephosphorylation of active receptors (Shi et al., 2004). I-Smads also form complexes with E3 ubiquitin ligases, such as Smad ubiquitination regulatory factor 1 or 2 (Smurf1 or Smurf2), resulting in the degradation of TGFβ receptors (Kim and Baek, 2018; Miller et al., 2018).

### Structure of Smad proteins

Smad structure accounts for differences in Smad function. Structurally, Smad proteins have a Mad Homology 1 (MH1) domain, separated by a flexible linker region from a MH2 domain (Shi et al., 1998; Macias et al., 2015). MH1 domains contain a nuclear localization signal and β-hairpin loop that mediates interactions with glycine cysteine-rich Smad-binding elements on DNA (Jonk et al., 1998; Shi et al., 1998), whereas MH2 domains interact with TGFβ receptors and mediate binding to other Smad proteins, transcription factors, and co-activators or co-repressors of transcription (Wu et al., 2001). Among the three regions, the greatest variability is observed within the linker region. The linker region of R-Smads contain phosphorylation sites for multiple kinases, such as CDKs and mitogen-activated protein kinases (MAPKs) (Massagué et al., 2005). Furthermore, within the linker region, both R-Smads and I-Smads, but not Smad4, have a proline-proline-x-tyrosine (PPXY) motif to bind to E3 ubiquitin ligases (Qin et al., 1999; Macias et al., 2015). Although MH1 and MH2 domains are highly conserved, there are some notable differences. I-Smads are missing the MH1 domain, therefore, cannot bind to DNA (Miyazawa and Miyazono, 2017). The MH2 domains of R-Smads have a β1-strand, L3 loop, and α-helix five structure that together mediates binding to TGFβRI or SARA (Shi et al., 1998; Wu et al., 2001; Macias et al., 2015). Although the structure of Smad2 and Smad3 are similar, there are notable differences. For instance, Smad2 has two inserts in its MH1 domain (Shi et al., 1998). One of these inserts, known as the E3 insert, was once believed to disrupt the β-hairpin loop, preventing Smad2 from binding DNA (Dennler et al., 1998; Dennler et al., 1999). Further analysis indicated that different conformations of the E3 insert regulate MH1 domain structure, which explains why in some instances Smad2 has been shown to bind to DNA (Aragón et al., 2019).

Although Smad4 is essential to many TGFβ-dependent changes in gene expression, Smad4 is not essential for R-Smad nuclear translocation nor is it necessary for some TGFβ-dependent transcriptional functions (Ten Dijke and Hill, 2004). Smad4 also performs TGFβ-independent functions that include silencing the expression of TGFβ target genes in T-lymphocytes (T-cells) (Igalouzene et al., 2022), upregulating genes that promote natural killer (NK) cell maturation (Wang et al., 2018), and tumour suppression by mediating Aurora A kinase degradation (Jia et al., 2014). Although the roles of Smad4 remain incompletely understood, Smad4 is the only Smad with a nuclear export signal and a Smad activation domain (SAD) within its linker region. The SAD region is recognized by the chromatin modifiers p300 and CREB-binding protein co-activators (Pouponnot et al., 1998). Although Smad4 SAD deletion cells are still able to bind p300 and CREB co-activators, these Smad4-p300 and Smad4-CREB complexes are unable to activate transcription (De Caestecker et al., 2000). In this manner, Smad4 contributes to the regulation of gene expression through p300 and CREB-binding protein co-activator complexes.

### Smad-independent TGFβ signalling

Smad-independent TGFβ signalling occurs through various pathways (Figure 2B) (Zhang, 2009). One involves the MAPK cascade *via* tumour necrosis factor receptor-associated factor 6 (TRAF6). Upon stimulation by TGFβ, TGFβRI associates with TRAF6, leading to lysine (K)63 polyubiquitination of this protein. K63-linked polyubiquitination provides a scaffold that subsequently recruits TGFβ-activated kinase 1 (TAK1), as well as TAK1-binding proteins. After TAK1-dependent phosphorylation, MAPK kinase 3/6 phosphorylates c-Jun amino-terminal kinase (JNK) and p38 MAPK. JNK and p38 MAPK translocate into the nucleus, where they phosphorylate several targets, including p53, activator protein 1 (AP1), E-twenty-six like-1 protein (ELK1), activating transcription factor 2 (ATF2), and cJun (Yamashita et al., 2008). These transcription factors regulate the expression of genes involved in apoptosis, inflammation, motility, development, cell-cell attachments, cell-ECM attachments, and proliferation (De Borst et al., 2006).

The protein kinase B (AKT) pathway is activated by TGFβRI phosphorylation of phosphoinositide 3-kinase (PI3K), which in turn activates AKT (Suwanabol et al., 2012). Downstream targets of AKT include mechanistic target of rapamycin (mTOR), a regulator of cell growth, proliferation, motility, survival, autophagy, transcription, and protein synthesis (Zhang et al., 2013). Additionally, AKT inhibits Forkhead box O (FoxO) transcription factors, which are important regulators of CDKs, survival, DNA repair, and T-cell activity (Zhang et al., 2005; Zhang et al., 2011).

Smad-independent TGFβ signalling also leads to modulation of small GTPase activity (Edlund et al., 2002). Specifically, TGFβRII can phosphorylate partitioning defective six homolog (Par6) (Ozdamar et al., 2005), whereas Ras homolog family member A (RhoA), and cell division control protein 42 (CDC42) activation relies on TGFβRI activity (Fleming et al., 2009; Kim et al., 2016). These proteins modulate cell-cell and cell-ECM attachments by regulating the function, stability, and organization of proteins essential to adherens and tight junctions. RhoA also promotes cell migration by inducing stress fiber formation (Warner et al., 2010; Nunes de Almeida et al., 2019). Stress fibers are contractile actomyosin bundles found in non-muscle cells composed of filamentous actin, α-actinin, and non-muscle myosin II filaments that may aid in cell movement (Hakkinen et al., 2011; Lehtimäki et al., 2021).

Tyrosine residues on the src homology domain containing protein A (ShcA) was also reported to be phosphorylated by TGFβRI (Lee et al., 2007). ShcA forms a complex containing growth factor receptor bound 2 (Grb2) and sons of sevenless (Sos) to activate Ras. The latter initiates downstream MAPK cascades that ultimately phosphorylates extracellular signal-regulated kinase (ERK) (Derynck and Zhang, 2003). ERK phosphorylates transcription factors, such as AP1 and ELK1, that regulate the expression of genes essential for cell-cell attachments, cell-ECM attachments, motility, differentiation, proliferation, and apoptosis (Zhang, 2009; Mu et al., 2012).

### TGFβ receptor endocytosis regulates signalling strength and duration

**FIGURE 3:**
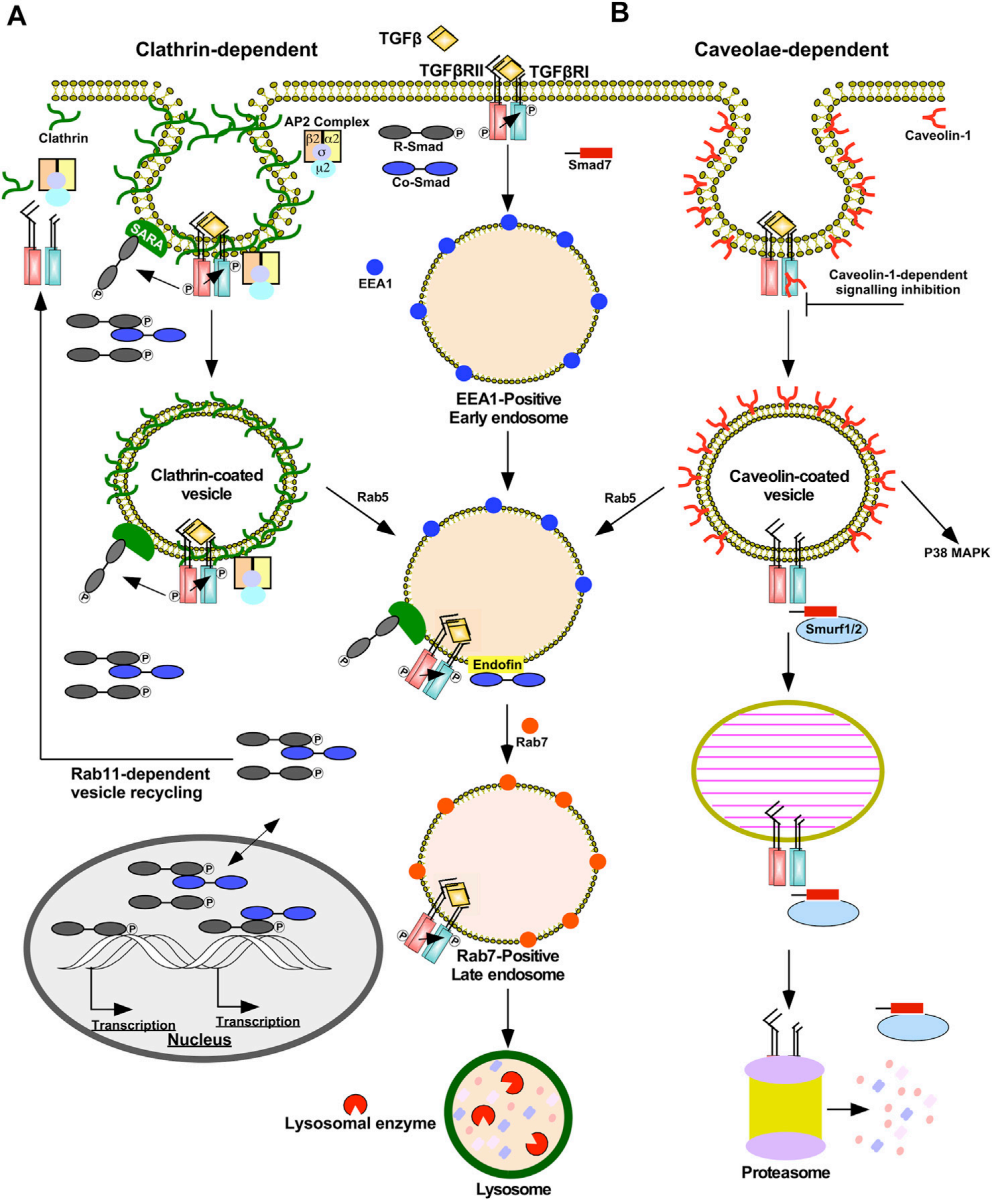
Clathrin- and caveolae-dependent endocytosis regulates the duration and strength of TGFβ signalling. **(A)** Clathrin-dependent receptor trafficking is mediated by triskelion shaped clathrin proteins (green). Clathrin tethers transforming growth factor-β (TGFβ) receptors to clathrin-coated pits *via* the β2 adaptin of the clathrin coat adaptor complex 2 (AP2). Clathrin-coated pits pinch off the plasma membrane to form clathrin-coated vesicles that fuse with early endosome membrane compartments by a Rab5-dependent process. In the presence of TGFβ, TGFβ receptors within clathrin-coated vesicles are active and phosphorylate downstream signalling molecules, such as Smads. Clathrin-coated pits and vesicles are enriched in Smad anchor for receptor activation (SARA) proteins that bind to R-Smads, which augments TGFβ signalling. Early endosomes bind to FYVE domain-containing proteins, such as endofin and SARA. Endofin enhances TGFβ signalling in early endosomes by tethering Smad4 to early endosomes. Clathrin-dependent receptor trafficking promotes R-Smad phosphorylation, which subsequently enters the nucleus with and without Smad4 to regulate transcription. The fates of the TGFβ receptors subjected to clathrin-dependent receptor trafficking involve recycling back to the plasma membrane in Rab11-positive vesicles or lysosomal degradation. Lysosomal degradation occurs after early endosomes mature into Rab7-positive late endosomes, which eventually fuse with lysosomes. **(B)** Caveolae-dependent receptor trafficking is facilitated by caveolin-1 proteins (red). Caveolae-coated vesicles are associated with dampening TGFβ signalling; however, non-canonical p38 MAPK signalling requires caveolae-coated vesicles. Caveolin-1 may bind to TGFβ receptor type I (TGFβRI) directly and attenuate its kinase activity. Caveolae-coated vesicles are enriched with Smad7-Smurf2 complexes that target TGFβ receptors to proteasome-dependent degradation. Prior to degradation, caveolae-coated vesicles may fuse with early endosomes in a Rab5-dependent manner or mature into caveolin-1-positive endosomes known as caveosomes.

Endocytosis of TGFβRI, TGFβRII, and TGFβ-TGFβRII complexes are mediated *via* clathrin- or caveolae-dependent mechanisms (Figure 3) (Le Roy and Wrana, 2005). Clathrin-dependent endocytosis allows TGFβ signalling to continue following receptor internalization and is associated with signal amplification (Yakymovych et al., 2018). Clathrin-coated pits sequester TGFβ receptors *via* the clathrin coat adaptor complex 2 (AP2) (Yao et al., 2002). AP2 is a hetero-tetramer that binds to clathrin and consists of four adaptins (β2, µ2, α, and σ2) (Kovtun et al., 2020). Unlike many receptors within the plasma membrane that bind to µ2-adaptin, TGFβ receptors directly bind to β2-adaptin (Yao et al., 2002). Next, several proteins facilitate budding and fission of clathrin-coated pits that are internalized as clathrin-coated vesicles. Clathrin-coated vesicles subsequently shed AP2 and fuse with the early endosome membrane compartment in a Rab5-dependent manner (Semerdjieva et al., 2008). Early endosome membrane compartments are enriched in phosphatidylinositol 3-phosphate (PI3P), which serve as recruitment sites for FYVE domain-containing proteins, such as early endosome antigen 1 (EEA1), endofin, and SARA (Lee et al., 2005). By associating with SARA on early-endosomal membranes, the R-Smads, Smad2/3, are poised to interact with TGFβ receptors (Itoh et al., 2002). Since regions involved in clathrin-dependent internalization are enriched in SARA, these routes of subcellular trafficking promote TGFβRI-dependent R-Smad phosphorylation (Macias et al., 2015). SARA also amplifies TGFβ signalling because SARA overexpression leads to endosomal swelling, which delays receptor recycling/degradation (Hu et al., 2002). In support of this, when the localization of SARA and EEA1-positive early endosomes was disrupted, there was a decrease in both TGFβ-induced Smad2 phosphorylation and Smad2 nuclear translocation (Tsukazaki et al., 1998; Hayes et al., 2002). Finally, endofin facilitates TGFβ signalling because it binds to TGFβRI and Smad4, which brings Smad4 in close proximity to phosphorylated R-Smads. Indeed, endofin knockdown reduced transcriptional responses to TGFβ and impaired TGFβ-dependent apoptosis (Chen et al., 2007). Therefore, clathrin-dependent trafficking of TGFβ receptors enables R-Smad phosphorylation in the early endosome and prolongs the duration in which ligands, receptors, and downstream signalling molecules are in close proximity. The early endosome is primarily responsible for sorting endocytosed TGFβ receptors, which may either recycle back to the plasma membrane in Rab11-positive vesicles (Yin et al., 2013) or be degraded in Rab7-positive late endosomes and lysosomes (Feng et al., 1995) (Figure 3A).

Caveolae are plasma membrane invaginations enriched with caveolin-1 that are localized in membrane rafts, plasma membrane subdomains rich in cholesterol and glycosphingolipids (Golub et al., 2004). Caveolin-positive vesicles may mature into or fuse with pre-existing caveosomes or early endosomes in a Rab5-independent or -dependent manner, respectively (Pelkmans et al., 2004). Caveolin-dependent endocytosis is associated with dampening and disrupting TGFβ signalling. Unlike clathrin-coated vesicles, SARA localizes away from membrane rafts and Smad7-Smurf2 complexes are commonly associated with caveolin-positive vesicles. Due to the association with Smad7-Smurf2, TGFβRII/TGFβRI complexes within caveolin-positive vesicles are targeted for proteasomal degradation (Guglielmo et al., 2003; Le Roy and Wrana, 2005). Caveolin-1 also has been shown to directly bind to TGFβRI following stimulation, which suppresses Smad2 phosphorylation possibly by antagonizing TGFβRI kinase activity (Razani et al., 2001) (Figure 3B). Caveolin-1 also disrupts TGFβ signalling through association with CD109, a TGFβ co-receptor. In the presence of ligands, CD109 promotes the localization of TGFβ receptors in caveolae and increases receptor degradation (Bizet et al., 2011). Indeed, after the TGFβRII/TGFβRI complexes are endocytosed in caveolin-positive vesicles, TGFβ signalling is inhibited (Guglielmo et al., 2003). However, the activation of some non-Smad signalling pathways, such as p38 MAPK, rely on the localization of TGFβ receptors in caveolae (Zuo and Chen, 2009).

In summary, the route of TGFβ receptor subcellular trafficking regulates signalling duration, strength, and receptor fate (McLean and Di Guglielmo, 2014). Although some TGFβ signalling occurs in the absence of receptor internalization, clathrin- or caveolae-dependent endocytosis can enhance or dampen TGFβ signal transduction pathways (Yakymovych et al., 2018).

### The role of the ubiquitin-proteasome pathway in TGFβ signalling

**FIGURE 4:**
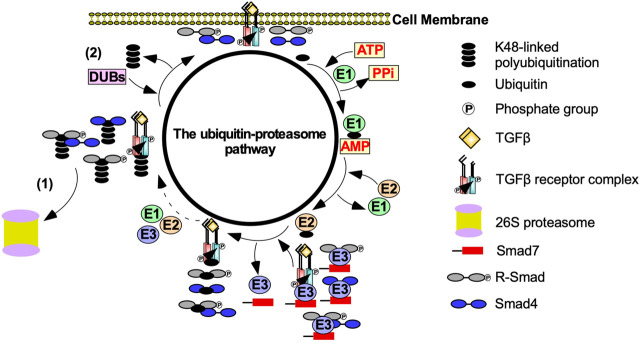
The effect of the ubiquitin-proteasome pathway on TGFβ signalling. Transforming growth factor-β (TGFβ) signalling is tightly regulated by the ubiquitin-proteasome pathway. After TGFβ binds to the TGFβ-receptors, the ubiquitin-proteasome pathway is activated to prevent uncontrolled TGFβ signalling. E1 activating enzymes hydrolases ATP to bind to ubiquitin. Ubiquitin is then transferred to an E2 conjugating enzyme. Smad7 binds to E3 ubiquitin ligases, which conjugates ubiquitin to TGFβ receptors, receptor Smads (R-Smads), Smad4, and R-Smad-Smad4 complexes. This process is repeated until TGFβ receptors, R-Smads, Smad4 or R-Smad-Smad4 complexes are polyubiquitinated. Polyubiquitinated components of the TGFβ pathway are then subject to (1) proteasome-dependent degradation or (2) the removal of the ubiquitin-linked chains mediated by deubiquitinating enzymes (DUBs).

The ubiquitin-proteasome pathway (UPP) also regulates the strength and duration of TGFβ signalling (Wang, 2003). The polyubiquitination of TGFβ receptors, R-Smads, and downstream effectors is dependent on E1 (activating), E2 (conjugating), and E3 (ubiquitin ligase) enzymes (Kim and Baek, 2018). E1 enzymes hydrolyze ATP to activate the C terminus of ubiquitin. Activated ubiquitin is then transferred to an E2 enzyme. E3 enzymes subsequently bind to E2-ubiquitin conjugates and transfers ubiquitin to K residues on TGFβ receptors, R-Smads or downstream effectors (Komander, 2009). K48-linked polyubiquitin chains target TGFβ receptors, R-Smads, and downstream effectors to 26S proteasomes, which are multi subunit proteases (Finley et al., 2016). Deubiquitinating enzymes decrease proteasome-dependent degradation by removing ubiquitin (Kim and Baek, 2018) (Figure 4). Although ubiquitination is important for proteasome-dependent degradation, it is also necessary to facilitate signalling (Adhikari et al., 2007). For instance, K63-linked polyubiquitination functions as a scaffold to recruit and activate protein kinase complexes (Yamashita et al., 2008). As previously discussed, ubiquitin ligases catalyze K63-linked polyubiquitin chains on TRAF6 to recruit TAK1 to facilitate Smad-independent TGFβ signalling (Landström, 2010).

Given that TGFβ signalling regulates a diverse set of cellular processes, modulating TGFβ signalling through a balance of ubiquitin ligase and deubiquitinating enzyme activity is important (Ten Dijke and Hill, 2004). By degrading TGFβ receptors, R-Smads, and downstream effectors, E3 ubiquitin ligases, protects cells from aberrant TGFβ signalling (Gao et al., 2009). However, there are numerous examples where ubiquitin ligases prolong TGFβ signalling. For instance, Smad2-Smurf2 complexes lead to the destruction of Ski-related protein N (SnoN) and Ski, which are protooncogenes that impede TGFβ signalling (Sun et al., 1999; Bonni et al., 2001). Arkadia, an E3 ubiquitin ligase, amplifies TGFβ signalling by ubiquitinating I-Smads (Koinuma et al., 2003). Paradoxically, if deubiquitinating enzymes remove K48-linked polyubiquitin chains on SnoN, Ski or Smad7, TGFβ signalling is dampened (Zhao et al., 2011). Therefore, ubiquitin ligases and deubiquitinating enzymes may both antagonize or promote TGFβ signalling depending on the function of the ubiquitinated protein.

## Mutations in genes involved in TGFβ signalling

Alterations in the TGFβ signalling pathway due to genetic mutations are the underlying cause of various hereditary congenital malformations, as well as diseases that arise later in life (Wang et al., 2012; Saito et al., 2018). Germline mutations impair embryonic development, whereas increased susceptibility to develop cancer is associated with somatic mutations (Harradine and Akhurst, 2006). The clinical consequences of mutations in the TGFβ signalling pathway are complex, because the tumour microenvironment and TGFβ signalling vary among patients and among different tissues within the same individual (Massagué, 2008).

### Germline mutations in the TGFβ signalling pathway

Genetically engineered mouse models with targeted inactivation of various TGFβ ligands have been generated to investigate the importance of TGFβ on development and viability (Glick, 2012). *Tgfb1* ^*−/−*^ mice can either succumb during mid-gestation as a result of vascular and hematopoiesis defects, or a few weeks after as a consequence of systemic inflammation (Shull et al., 1992; Kulkarni et al., 1993; Dickson et al., 1995). Death occurs shortly before, during or within minutes of birth in *Tgfb2* ^*−/−*^ mice, due to impaired cardiovascular function. These animals exhibit cardiac, craniofacial, limb, eye, inner ear, and urogenital defects (Sanford et al., 1997; Dünker and Krieglstein, 2002). *Tgfb3* ^*−/−*^ mice exhibit cleft palates that interfere with feeding, eventually resulting in death (Dünker and Krieglstein, 2002; Aluwihare et al., 2009). The majority of *Smad*-null mice die *in utero*, indicating that Smad proteins are required for proper embryonic development as previously reviewed (Datto and Wang, 2000). Specifically, *Smad2* ^*−/−*^ and *Smad4* ^−/−^ mice die early in embryogenesis, due to defects in the organization of the primitive germ layers and extensive mesodermal defects (Nomura and Li, 1998; Chu et al., 2004). *Smad3* ^*−/−*^ mice are viable, but exhibit impaired local inflammatory responses and accelerated wound healing (Ashcroft et al., 1999; Ling and Robinson, 2002).

In patients, familial juvenile polyposis, which increases the risk of gastrointestinal cancer, is correlated with *SMAD4* mutants that produce truncated proteins with a loss or partial loss of function (Howe et al., 1998; Johansson et al., 2015). Although juvenile polyposis patients have been screened for *SMAD2* and *SMAD3* mutations, only *SMAD4* germline mutants are identified as an underlying cause of juvenile polyposis (Bevan et al., 1999). However, screening colorectal adenoma patients revealed that mutations to the *SMAD4* loci are rare (Lipton et al., 2003). *SMAD4* mutations in patients with juvenile polyposis syndrome may also develop hereditary hemorrhagic telangiectasia, which results in abnormal vascular structures (Heald et al., 2015).

### Somatic mutations in the TGFβ signalling pathway

Frameshift and missense mutations in *TGFBRI* are common in several tumour types (Moore-Smith and Pasche, 2011). For example, the TGFBRI*6A mutation in exon one is a loss of three Alanine residues in a 9-Alanine repeat region that increases cancer susceptibility associated with impaired anti-proliferative TGFβ signalling (Liao et al., 2010). Inactivating mutations in *TGFBR2* are frequently present in tumours that exhibit microsatellite instability (Vincent et al., 1996), such as those found in subsets of colon carcinomas, which express truncated mutant forms of TGFβR2 (Ogino et al., 2007). *SMAD4* is the most common Smad family gene mutated in malignant tumours (Sarshekeh et al., 2017). Inactivating *SMAD4* mutations have been found in approximately 50% of pancreatic adenocarcinomas (Howe et al., 1998), 20% of colorectal carcinomas (Chu et al., 2004), and 5% of head and neck squamous cell carcinomas (Lin et al., 2019a). Smad4 mutations also correlate with tumour formation (Lin et al., 2019b) and may predict poor prognosis and aggressive tumour phenotypes (Fang et al., 2021). For instance, mice with conditional targeted inactivation of *Smad4* in the oral epithelium developed spontaneous squamous cell carcinomas (Bornstein et al., 2009). Although somatic mutations of the TGFβ pathway may promote tumour formation, similar mutations in cancerous cells that rely on TGFβ can decrease tumour growth (Pino et al., 2010). Since somatic mutations of the TGFβ pathway may promote or block tumourigenesis depending on the stage of the disease, this is important to bear in mind when assessing the benefits and risks of using TGFβ signalling inhibitors in cancer treatment (Khoshakhlagh et al., 2019).

## TGFβ signalling in tumourigenesis

Cells escape the tumour suppressing arms of TGFβ signalling through mutations that impede specific TGFβ pathways or abnormalities in processes that dampen TGFβ signalling (David and Massagué, 2018). Functional inactivation of the tumour suppressing arms of TGFβ signalling can contribute to carcinogenesis through various mechanisms (Massagué, 2008; David and Massagué, 2018). Major mechanisms that contribute to the pro-tumourigenic effects of TGFβ include inhibition of immune function, activation of angiogenesis/lymphangiogenesis, and the initiation of EMT (Ferrari et al., 2009; Flavell et al., 2010; Batlle and Massagué, 2019).

### Inhibition of anti-cancer immune responses

As prolonged activation of the immune system can induce inflammation and tissue damage, the immune system is modulated through inhibitory mechanisms (Sitkovsky and Ohta, 2005). Cells in the tumour and its microenvironment benefit from these immunological safeguards by producing excessive amounts of immunosuppressive cytokines, such as TGFβ (Flavell et al., 2010; Batlle and Massagué, 2019). TGFβ inhibits many components of both the innate and adaptive immune systems, which creates an environment favourable for tumour growth (Moo-Young et al., 2009).

**FIGURE 5:**
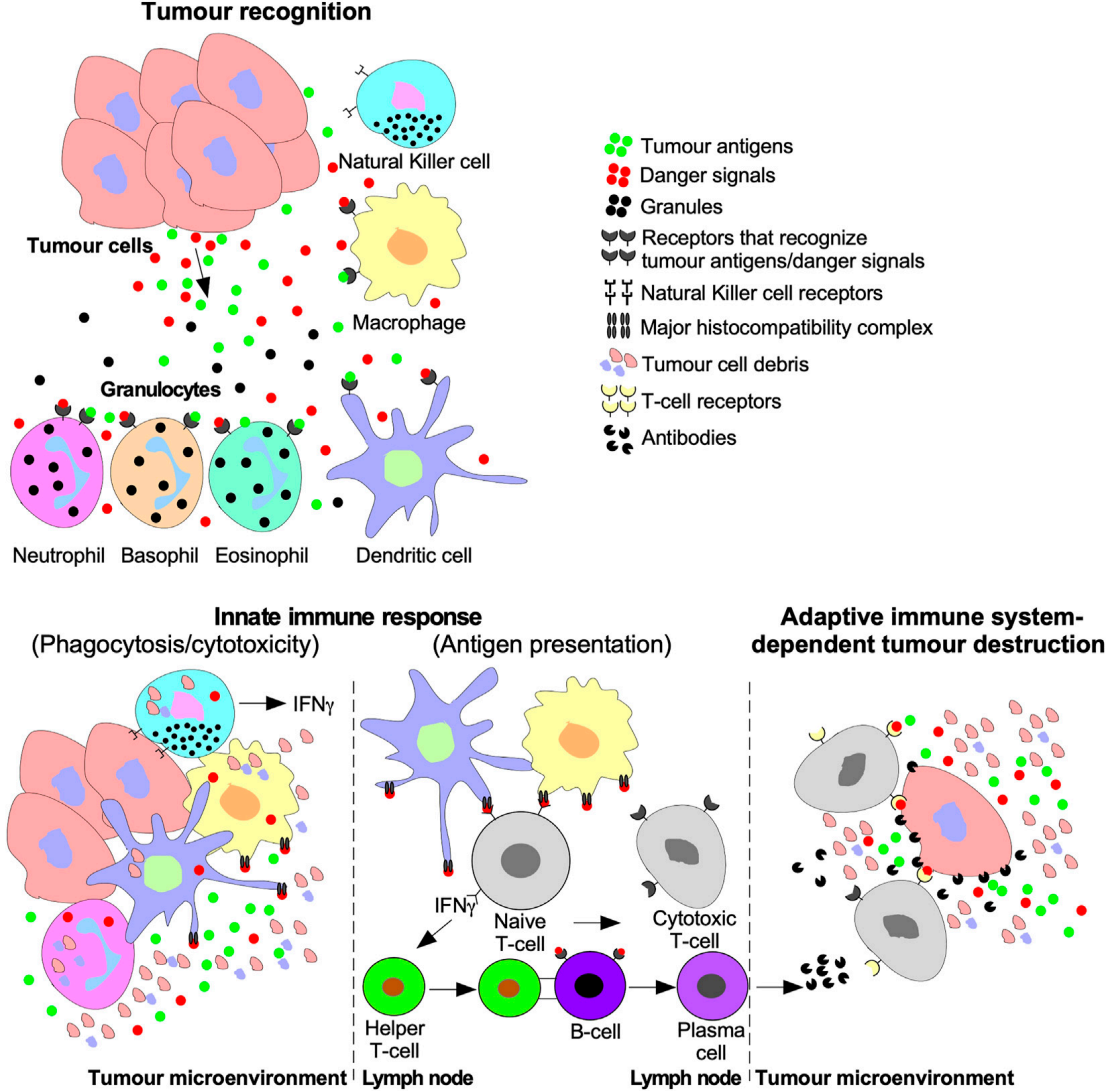
Tumour recognition and destruction mediated by the innate and adaptive immune systems. Tumour cells release antigens and danger signals that serve as a chemotactic gradient to recruit cells of the innate immune system ((Natural Killer (NK) cells, macrophages, dendritic cells, and granulocytes (neutrophils, basophils, and eosinophils)). Cells of the innate immune system may destroy tumours using cytolytic/phagocytic functions or activate the adaptive immune system. The adaptive immune system is activated by humoral signals, such as interferon-γ (IFNγ), which is released by NK cells, dendritic cells, and macrophages. Furthermore, antigen presenting macrophages and dendritic cells deliver tumour antigens using the major histocompatibility complex to Naive T-lymphocytes (T-cells) or B-lymphocytes (B-cells). Naive T-cells are stimulated to differentiate into Cytotoxic T-cells and Helper T-cells. B-cell differentiation into cytotoxic antibody-producing plasma cells is triggered by B-cell receptors binding to Helper T-cells or tumour antigens. The adaptive immune system facilitates tumour destruction *via* Cytotoxic T-cells releasing enzymes into tumour cells or antibodies produced by plasma cells.

**FIGURE 6:**
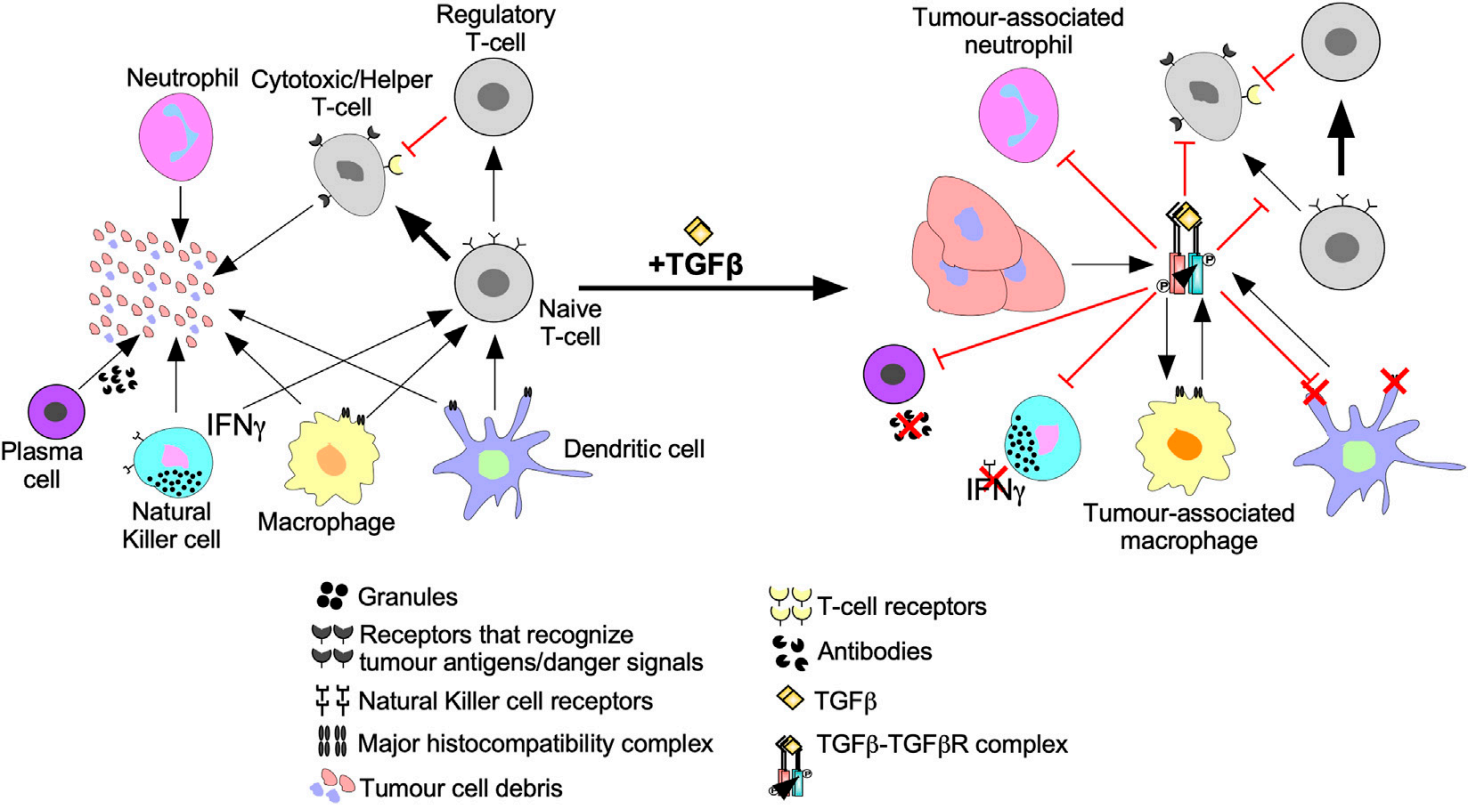
The inhibition of anti-cancer immune responses by TGFβ. In the absence of immunosuppressive cytokines, cells of the innate and adaptive immune system destroy tumour cells as described in Figure 7. However, the addition of transforming growth factor-β (TGFβ) suppresses tumour recognition and cytotoxic functions of the innate and adaptive immune systems. For instance, TGFβ suppresses antigen presenting function of macrophages, neutrophils, and dendritic cells by downregulation the major histocompatibility complex. TGFβ decreases Natural Killer cell receptors, which spares tumour cells from Natural Killer cell-mediated destruction. TGFβ dampens immune cell recruitment by disrupting interferon-γ (IFNγ) production in Natural Killer cells, dendritic cells, and macrophages. TGFβ induces macrophages and neutrophils to differentiate into tumour-associated macrophages and tumour-associated neutrophils, respectively, which augment tumourigenesis. TGFβ disrupts B-cell differentiation into plasma cells and attenuates antibody production. TGFβ also alters Naive T-cell differentiation to favour tumour promoting Regulatory T-cells instead of Cytotoxic T-cells or Helper T-cells that mediate tumour cell destruction. Regulatory T-cells promote tumourigenesis by suppressing Cytotoxic T-cell function.

Tumour cells are targeted for destruction by cells of the innate immune system, which include monocytes, macrophages, dendritic cells, neutrophils, basophils, eosinophils, and NK cells (Gajewski et al., 2013). Through phagocytosis, macrophages, neutrophils, and dendritic cells engulf tumour cell debris and tumour cells missing essential cell surface proteins or expressing danger signals (Chan and Housseau, 2008; Sarode and Sarode, 2014; Zhou et al., 2021). Macrophages, neutrophils, and dendritic cells also attach antigens to their major histocompatibility complexes (MHCs) to activate T- and B- lymphocytes (T- and B-cells) of the adaptive immune system (Figure 5) (Gajewski et al., 2013). The effects of TGFβ on dendritic cells include interference with antigen presenting activity, immobilization, and upregulation of TGFβ production, creating a positive feedback loop to maintain a decrease in immune responses against the tumour (Esebanmen and Langridge, 2017). Furthermore, by interfering with dendritic cell antigen presenting activity, TGFβ blocks naive T-cell and B-cell differentiation into anti-tumour phenotypes (Liu et al., 2018). TGFβ within the tumour microenvironment may manipulate macrophages and neutrophils to differentiate into phenotypes that contributes to tumour growth rather than destroy tumour cells. These macrophages and neutrophils are typically referred to as tumour-associated macrophages (TAMs) and tumour-associated neutrophils (TANs), respectively (Fridlender et al., 2009; Danhier et al., 2017). TGFβ-recruited TAMs can phagocytose antigen-containing particles prior to their recognition by dendritic cells. Therefore, TAMs suppress the antigen presenting abilities of dendritic cells, hindering activation of the adaptive immune system (Liu et al., 2018; Batlle and Massagué, 2019). TGFβ recruited TANs have decreased cytotoxicity and secrete extensive quantities of MMPs to free TGFβ from large latent TGFβ complexes, which increases the concentration of active TGFβ ligands in the tumour microenvironment, contributing to a positive feedback loop (Figure 6) (Germann et al., 2020).

NK cells are specialized leukocytes that do not rely on MHCs or humoral signals to recognize tumour cells (Abel et al., 2018). Instead, NK cells recognize tumour cells using cell surface receptors. Upon binding to tumour cells, NK cells release interferon-γ (IFNγ) into the tumour microenvironment and cytolytic antibodies directly into the tumour cell (Castro et al., 2018). Thus, NK cells eliminate tumour cells by triggering an antibody-dependent cell-mediated cytotoxic response and activate other leukocytes using IFNγ (Figure 5) (Abel et al., 2018). TGFβ blocks NK cell-mediated adaptive immune system activation by downregulating the transcription factor T-bet, leading to reduced IFNγ expression (Hayashi et al., 2003; Mohammadzadeh et al., 2014). The TGFβ-dependent loss of IFNγ decreases the activity of leukocytes, downregulates antigen presenting MHCs in antigen presenting leukocytes, and impedes chemotaxis (Castro et al., 2018). TGFβ also downregulates NK receptors responsible for recognizing and destroying tumour cells (Figure 6) (Castriconi et al., 2003).

Like the innate immune system, the adaptive immune system facilitates tumour cell death using humoral immunity and cell-mediated immunity. Cell-mediated immunity and humoral immunity is facilitated by T-cells. Following antigen presentation, naive T-cells differentiate into effector T-cells, such as cytotoxic T-cells and helper T-cells (Fazilleau et al., 2009; Farhood et al., 2019). Cytotoxic T-cells specifically eliminate cells expressing the antigen presented whereas helper T-cells release humoral signals to activate other leukocytes (Figure 5) (Belardelli and Ferrantini, 2002; Fazilleau et al., 2009). In tumour microenvironments with elevated TGFβ levels, decreased numbers and limited anti-tumour cytolytic activity of cytotoxic T-cells have been observed, through mechanisms that include induction of T-cell apoptosis (Thomas and Massagué, 2005; Flavell et al., 2010; Liu et al., 2018). TGFβ also disrupts T-cell anti-tumourigenic activity by upregulating genes that promote naive T-cell differentiation into less cytotoxic phenotypes, such as Tregs (Figure 6) (Zhang et al., 2018). Plasma cells are adaptive immune system cells that mediate humoral immunity. Upon antigen presentation, B-cells differentiate into plasma cells that produce antibodies to eliminate tumour cells (Figure 5) (Kurosaki et al., 2015). TGFβ attenuates the anti-tumourigenic capacity of B-cells by interfering with their differentiation into plasma cells, antibody production, and proliferation (Figure 6) (Schwartz et al., 2016).

### Activation of angiogenesis and lymphangiogenesis

**FIGURE 7:**
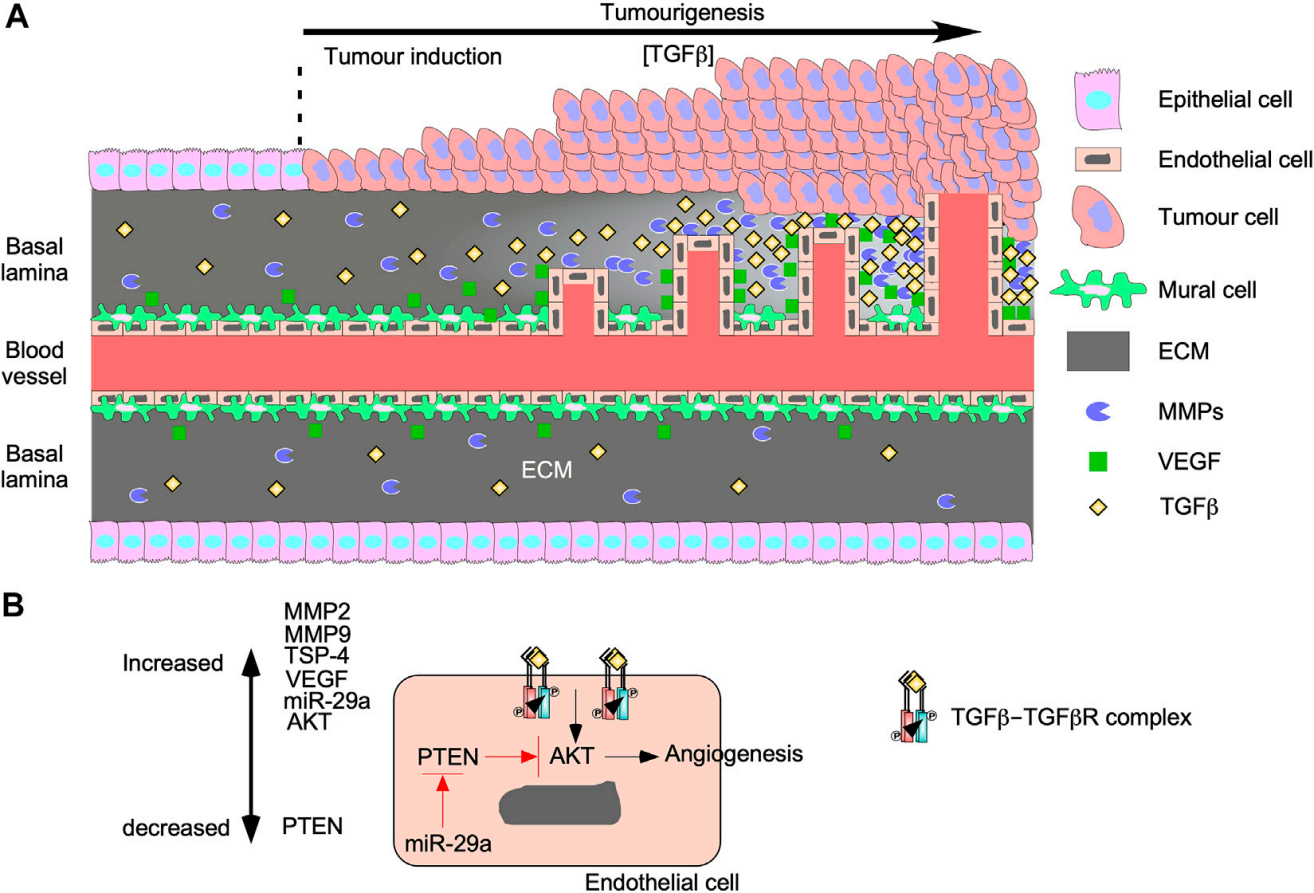
TGFβ augments tumourigenesis by inducing angiogenesis. **(A)** As tumours grow, the concentration of transforming growth factor-β (TGFβ) in the tumour microenvironment increases. TGFβ upregulates genes involved with proliferation and migration in mural cells, which results in endothelial cell migration and leaky vessels. TGFβ binds to TGFβ receptors on endothelial cells to upregulate vascular endothelial growth factor (VEGF) and matrix metalloproteinases (MMPs). Both proteins are secreted into the basal lamina and increase proportionally to TGFβ. VEGF binds to endothelial cells and stimulates proliferation and migration. MMPs breakdown proteins in the basal lamina to remodel the extracellular matrix (ECM) to carve out space for vessel formation. The new vessels grow and become more organized as time passes. **(B)** TGFβ binds to TGFβ receptors on endothelial cells and upregulate MMP2, MMP9, thrombospondin-4 (TSP-4), microRNA-29s (miR-29a), VEGF, and protein kinase B (AKT). miR-29a blocks the translation of phosphatase and tensin homolog (PTEN), which is a known AKT inhibitor. Since the AKT pathway has been linked to angiogenesis, TGFβ signalling may induce angiogenesis through the AKT pathway.

Angiogenesis promotes tumour growth and invasion because as tumours grow, blood carrying oxygen and nutrients is blocked from reaching interior tumour cells (Nishida et al., 2006). To bypass this, tumour microenvironments are enriched with cytokines, such as TGFβ, that alter cellular processes within endothelial cells and mural cells to generate new vessels (Figure 7A) (Ferrari et al., 2009). The effects of TGFβ on angiogenesis, endothelial cells, and on mural cells are complex. Although in normal vessels TGFβ supports vascular development by recruiting mural cells toward endothelial cells (Walshe et al., 2009), TGFβ in tumour vasculature induces the differentiation of endothelial cells into mural cells (Hirschi et al., 2003). Then, mural cells secrete angiogenic factors and form defective interactions with endothelial cells resulting in disorganized vasculature (Sun et al., 2021). In endothelial cells, binding of TGFβ to TGFBRII leads to the activation of two distinct type I receptors: endothelial cell-specific activin receptor-like kinase 1, which signals through Smad1/5/8, as well as the ubiquitous TGFβRI, which signals through Smad2/3 (COLLETTA et al., 1988; Goumans et al., 2002; Mallet et al., 2006; Ito et al., 2009). Smad1/5/8 signalling induces endothelial cell proliferation and migration (Ray et al., 2010), whereas Smad2/3 signalling induces endothelial cell differentiation into mesenchymal-like mural cells (Hirschi et al., 2003; Jiang et al., 2018). TGFβ can promote angiogenesis through TGFβRI, but inhibits growth factor-induced endothelial sprouting/branching through mechanisms that involve cross-talk with Notch-activated pathways (Mallet et al., 2006; Aspalter et al., 2015). In mural cells and endothelial cells, TGFβ also induces Smad-dependent expression of vascular endothelial growth factor (VEGF), thrombospondin-4 (TSP-4), MMPs, microRNA-29a, and other genes that stimulate endothelial cell proliferation and migration (Massagué, 2008; Ferrari et al., 2009).

VEGF enhances endothelial cell migration, proliferation, and resistance to apoptosis (Ferrari et al., 2009; Suzuki et al., 2012) by activating two tyrosine kinase VEGF receptors (VEGFR1 and VEGFR2). VEGFR1 activation is involved with migration whereas VEGFR2 activation regulates proliferation and survival (Wang et al., 2017). Interestingly, TGFβ activates apoptosis, which suggests that VEGF and TGFβ have opposing roles on endothelial cell survival. However, many studies suggest that pro-apoptotic TGFβ signalling is necessary for angiogenesis because it ensures less branching and increases vasculature organization (Haque and Morris, 2017). Furthermore, TGFβ upregulates ECM remodelling proteins in endothelial cells, such as TSP-4 and MMPs (Tirino et al., 2013; Muppala et al., 2017). By a Smad3-dependent mechanism, TGFβ activates post-translation processes that increase TSP-4 protein levels (Muppala et al., 2017). The importance of TSP-4 on endothelial cell proliferation and migration during angiogenesis was verified when TGFβ-induced angiogenesis was attenuated in *Tsp-4* ^−/−^ mouse models (Muppala et al., 2017). Additionally, TGFβ upregulates the expression of MMP2 and MMP9 in endothelial cells and cells of the tumour microenvironment, thus facilitating ECM remodelling and releasing ECM-sequestered cytokines (Yu and Stamenkovic, 2000). Therefore, MMPs play a role in TGFβ-mediated angiogenesis by releasing latent TGFβ from LAP and LTBP (Tatti et al., 2008) as well as generating the space required for endothelial cell migration, proliferation, and microvessel formation (Park et al., 2018). Finally, microRNA-29a silences phosphatase and tensin homolog (PTEN) RNA expression (Wang et al., 2013), leading to increased AKT pathway activity and activation of TGFβ-induced angiogenesis (Chen et al., 2020). Since blocking PTEN activity increases the activity of the AKT pathway (Chen et al., 2020), the Smad-independent PI3K/AKT TGFβ signalling pathway may play a major role in TGFβ-induced angiogenesis (Figure 7B).

Tumour cells primarily metastasize through the lymphatic system due to the thinner walls and increased permeability of lymphatic vessels, relative to blood vasculature (Chaffer et al., 2016). Furthermore, cancer cells may drain directly into the lymphatic system if they break free from tumours (Karlsson et al., 2017). Two mechanisms for TGFβ contribution to metastasis through the lymphatic system have been proposed. Due to the greater representation of leukocytes in the lymphatic system, lymph node metastasis requires immune suppression (Liu and Cao, 2016). Therefore, the inhibitory effects of TGFβ on leukocytes present in the lymphatic system may promote tumour cell survival and increases dissemination (Liu and Cao, 2016). Additionally, Smad-dependent and -independent TGFβ signalling induces lymphangiogenesis, formation of new lymphatic vessels from pre-existing lymphatic vessels (García-Caballero et al., 2017), by upregulating VEGF-C, which in turn promotes growth, proliferation, migration, and survival of endothelial cells bordering lymphatic vessels (Pak et al., 2019). Cells of the tumour microenvironment that respond to TGFβ, such as TAMs, may also mediate lymphangiogenesis *via* a VEGF receptor 3-dependent process (Alishekevitz et al., 2016).

### Epithelial-mesenchymal transition (EMT)

**FIGURE 8:**
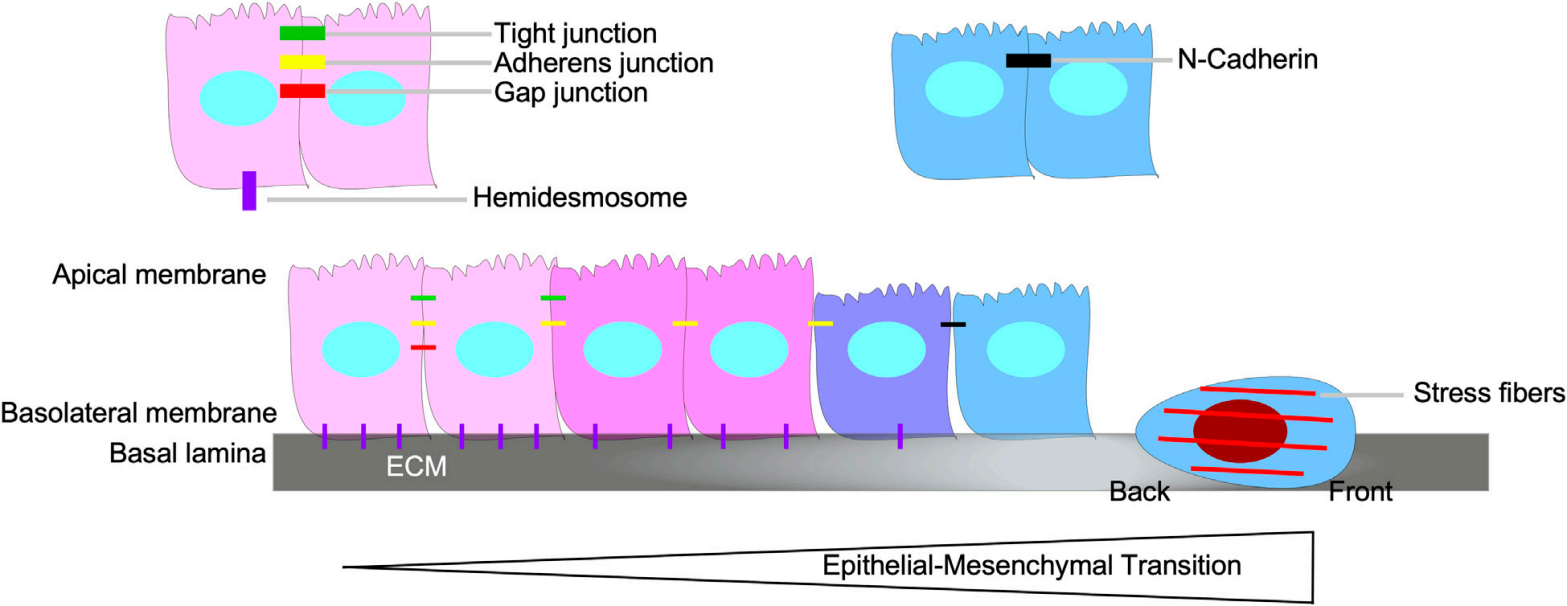
Epithelial-mesenchymal transition. Epithelial-mesenchymal transition (EMT) is the biological process of an epithelial cell loses its epithelial properties, such as apical/basolateral polarity, tight junctions, gap junctions, adherens junctions, and hemidesmosomes, and develop mesenchymal properties, which includes the capacity to breakdown the basal lamina, assert back/front polarity, spindle-shaped morphology, induce stress fiber formation, and N-Cadherin-dependent cell-cell attachments.

Epithelial-mesenchymal transition (EMT), a biological process whereby cells of epithelial origin acquire characteristics of mesenchymal cells, is essential for embryogenesis and wound healing (Tan et al., 2015; Chaffer et al., 2016). EMT is involved in the ability of carcinoma cells to acquire motile and invasive phenotypes, thus contributing to tumour progression and metastasis (Craene and Berx, 2013). During EMT, there is a loss of epithelial properties, such as apical/basolateral polarity, cytoskeleton polarization, cell-cell adhesions (adherens junctions, tight junctions, and gap junctions), and attachment to the basal lamina. Subsequently, the cells acquire spindle-shaped morphology, transient focal point cell-cell attachments, lamellipodia/filopodia formation, front-back polarity, stress fibers, and increased motility (Figure 8) (Chaffer et al., 2016; Karlsson et al., 2017).

**FIGURE 9:**
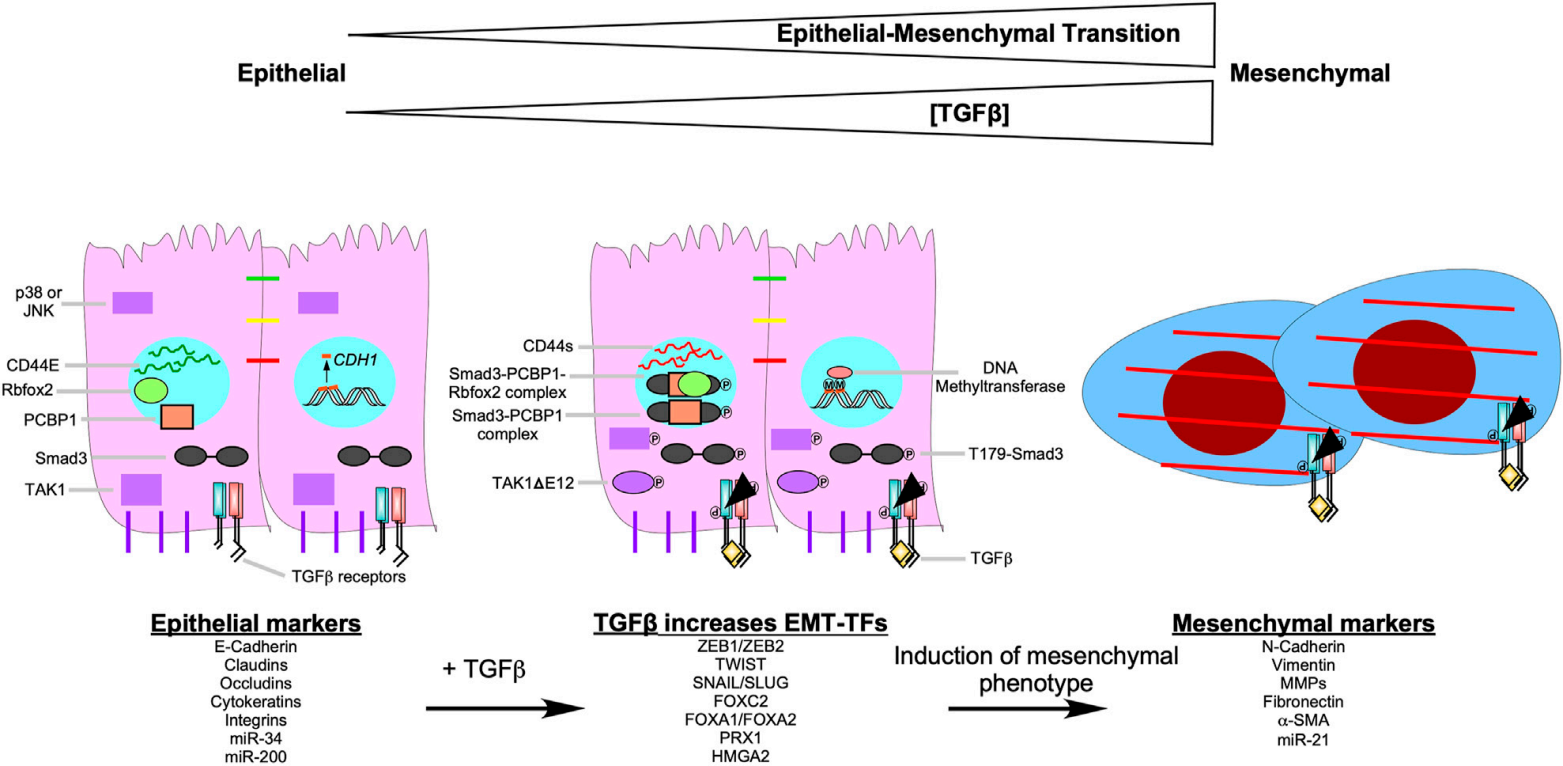
TGFβ signalling pathways that induce epithelial-mesenchymal transition. As the concentration of transforming growth factor-β (TGFβ) increases, the epithelial-mesenchymal transition (EMT) program becomes more pronounced. After TGFβ binds to the TGFβ receptors, it upregulates EMT-transcription factors (EMT-TFs), such as Snail Family Transcriptional Repressor one and 2 (SNAIL/SLUG), Zinc Finger E-box Binding Homeobox one and 2 (ZEB1/ZEB2), Twist-related Protein 1 (TWIST1), Forkhead box C2 (FOXC2), Forkhead box A1 (FOXA1), Forkhead box A2 (FOXA2), Paired-related Homeobox 1 (PRX1), and High Mobility Group AT-hook 2 (HMGA2). EMT-TFs downregulate epithelial markers ((E-Cadherin, claudins, occludins, cytokeratins, integrins, microRNA (miR)-34, and miR-200)) and upregulate mesenchymal markers ((N-Cadherin, vimentin, matrix metalloproteinases (MMPs), fibronectin, α-smooth muscle actin (α-SMA), and miR-21)). TGFβ induces EMT by increasing DNA methyltransferase activity. In the presence of TGFβ, DNA methyltransferase methylates (M) the promoters of epithelial genes, such as *Cadherin 1*(*CDH1*). Also, when TGFβ receptor type I phosphorylates Smad3 at threonine 179 (T179-Smad3), it may associate with the RNA-binding protein poly (RC) binding protein 1 (PCBP1). Smad3-PCBP1 complexes alter CD44 splicing from CD44E, which is found in epithelial cells, to CD44s. CD44s splice variants modulate cell-cell adhesion to promote EMT. The Smad3-PCBP1 complex associated with Rbfox2 that mediates alternative splicing of TGFβ-activated kinase 1 (TAK1) to favour TAK1ΔGlu 12 (TAK1ΔE12) variants. TAK1ΔE12 is constitutively active, which leads to the constitutive phosphorylation of p38 MAPK (p38) and cJun N-terminal Kinase (JNK). P38 and JNK upregulate genes that promote EMT.

The profound phenotypical and morphological characteristics observed during EMT are amplified by signals that tumour cells receive from the tumour microenvironment, such as TGFβ (Kawata et al., 2012). TGFβ contributes to the initiation of the EMT program, *via* transcription-dependent and -independent mechanisms (Gunaratne and DiGuglielmo, 2013; Tirino et al., 2013; Ganesan et al., 2016; Tripathi et al., 2019). TGFβ upregulates various EMT-transcription factors (SNAIL, SLUG, TWIST, ZEB1, ZEB2, FOXC2, FOXA1, FOXA2, PRX1, and HMGA2), which decrease the expression of epithelial genes, whilst increasing that of mesenchymal genes (Figure 9) (Barrallo-Gimeno and Nieto, 2005; Kokudo et al., 2008; Kume, 2008; Miyazono, 2009; Xu et al., 2009; Mikheeva et al., 2010; Lee and Yutzey, 2011; Wu et al., 2011; Kaufhold and Bonavida, 2014; Ganesan et al., 2016; Niu et al., 2016; Katsura et al., 2017; Vu and Datta, 2017; Maturi et al., 2018; Atala, 2019; Stemmler et al., 2019)**.** For example, SNAIL, SLUG, and ZEB1 downregulate the expression of E-Cadherin, a protein required for strong adherens junctions observed in epithelial cells, whereas TWIST upregulates the expression of N-Cadherin, a mesenchymal protein that forms weak transient cell-cell interactions (Barrallo-Gimeno and Nieto, 2005; Mikheeva et al., 2010; Dhasarathy et al., 2011; Lee and Yutzey, 2011; Kaufhold and Bonavida, 2014; Maturi et al., 2018). An in-depth analysis of genes targeted by EMT-transcription factors that mediate the transition of epithelial to mesenchymal phenotypes are outlined in previous reviews (Wrana, 2013; Batlle and Massagué, 2019).

TGFβ can promote EMT through non-canonical, Smad3-dependent regulation of RNA splicing. Phosphorylation of Smad3 on Thr179, subsequent to TGFβ receptor stimulation, impairs binding to Smad4 and to DNA (Gao et al., 2009; Inui et al., 2011; Tang et al., 2011), but induces Smad3 association with the RNA-binding protein poly (RC) binding protein 1 (PCBP1) in the nucleus (Tripathi and Zhang, 2017). The Smad3-PCBP1 species catalyzes alternative splicing of myriad transcripts involved in EMT, including RNAs encoding the CD44 glycoprotein, which modulates cell-cell adhesion (Ponta et al., 2003). Multiple CD44 splice variants exist. CD44E is preferentially expressed in normal epithelial cells, whereas the mesenchymal isoform CD44s is ubiquitous. In epithelial carcinoma cells, Smad3-PCBP1 complexes induce a splicing switch from CD44E to CD44s, resulting in activation of EMT and invasion (Thomas and Massagué, 2005). Similarly, complex formation between Smad3, PCBP1, and the RNA-binding protein Rbfox2 mediates expression of the alternative TAK1 splice variant TAK1ΔGlu 12 (TAK1ΔE12) (Braeutigam et al., 2014). TAK1ΔE12 is constitutively active, which means downstream signalling kinases, such as p38 MAPK and JNK, are constitutively phosphorylated (Yamashita et al., 2008; Tripathi et al., 2019). Transcription factors regulated by p38 MAPK and JNK are involved with upregulating genes that promote proliferation and EMT (Figure 9) (Zhao et al., 2017).

Finally, TGFβ can also promote EMT by upregulating DNA methyltransferases, which hypermethylate promoters of various genes involved in the regulation of the cell cycle, apoptosis, cell-cell attachments, ECM production, and cell movement (Lu et al., 2017). For example, in ovarian carcinoma cells, reduced transcription of *CDH1,* which encodes E-Cadherin, is associated with hypermethylation in the presence of TGFβ (Figure 9) (Cardenas et al., 2014).

Similar to EMT, endothelial-mesenchymal transition (EndMT) occurs when endothelial cells lose tight junctions and downregulate various endothelial cell markers, such as VE-Cadherin, to acquire mesenchymal properties, including expression of α-smooth muscle actin and N-Cadherin (Hong et al., 2018). EndMT is important during cardiac development and wound healing, and is believed to be an important contributor to certain pathologies (Lin et al., 2012). EndMT has been described in cardiovascular pathologies, such as atherosclerosis, cardiac fibrosis, and pulmonary hypertension (Jimenez and Piera-Velazquez, 2016). Recently, evidence has emerged that some cancer-associated fibroblasts (CAFs) have an endothelial origin (Zeisberg et al., 2007). These CAFs express α-smooth muscle actin and type I collagen, which are markers associated with excessive scarring and ECM remodelling (Yeon et al., 2018). A pathway linking TGFβ to EndMT involves TGFβ-mediated upregulation of SNAIL, which in turn induces downregulation of VE-Cadherin (Platel et al., 2019). Additionally, when TGFβ-dependent ERK phosphorylation was blocked, TGFβ-dependent EndMT was attenuated (Wylie-Sears et al., 2014).

There are several factors involved with TGFβ-dependent EMT/EndMT regulation. First, the chromatin structure and epigenetics of a cell dictate if SNAIL and other transcription factors can access genes subject to their regulation (Millanes-Romero et al., 2013; Kaufhold and Bonavida, 2014). Second, miRNAs block the expression of EMT/EndMT-transcription factors. For instance, microRNA-34 and microRNA-200 prevent the translation of SNAIL and ZEB1, respectively (Chaffer et al., 2016; Imani et al., 2017; Title et al., 2018). Finally, each cell type has different intracellular signalling configurations. Therefore, the rate in which different cell types conduct Smad-dependent or -independent signalling is not the same (Wu et al., 2016). In conclusion, cells that upregulate microRNAs that block EMT/EndMT-transcription factor translation, contain DNA methylation in the promoters of genes regulated by EMT/EndMT-transcription factors, and favour tumour suppressive TGFβ pathways are less likely to undergo TGFβ-dependent EMT/EndMT.

## The relationship between autophagy and the tumour promoting properties of TGFβ

Immunosuppression, increased angiogenesis, and EMT are the most widely studied mechanisms whereby TGFβ promotes tumourigenesis. However, the pro-tumourigenic activity of TGFβ likely includes additional biological processes, such as autophagy (Suzuki et al., 2010). Autophagy, Greek for self-devouring, is a catabolic process where cells degrade and recycle their own macromolecules and organelles primarily *via* lysosomes (Kaur and Debnath, 2015). Autophagy is essential for recycling the building blocks of lipids, carbohydrates, and proteins as well as eliminating invading pathogens, protein aggregates, and damaged organelles (Bernard and Klionsky, 2013). Although autophagy is primarily facilitated by lysosomes, which are acidic organelles that contain luminal degradative hydrolases, other acidic vesicles, such as late endosomes, contribute to autophagic degradation (Lawrence and Zoncu, 2019).

The idea that TGFβ-dependent tumourigenesis may rely on autophagy is supported by the extensive roles that autophagy plays in tumour development, maintenance, and metastasis (Mathew et al., 2007). Similar to TGFβ, the tumour regulatory consequences of autophagy are context dependent, as autophagy can result in either tumour suppression or promotion, depending on the stage of tumour development (Kiyono et al., 2009; Glick et al., 2010). In non-cancerous tissues, autophagy functions as a homeostatic safeguard by removing protein aggregates, damaged organelles, and other metabolic stressors, all of which protects against neoplastic transformation (Mathew et al., 2009; Klionsky et al., 2016). However, autophagy participates in the survival of established tumour cells under conditions of hypoxia, oxidative damage, metabolic stress, and starvation. Furthermore, cancer cells with elevated rates of autophagy tend to grow more rapidly and are prone to metastasize (Kiyono et al., 2009; Rebecca and Amaravadi, 2016; Alizadeh et al., 2018). Autophagy has been linked to EMT, MMP secretion, angiogenesis, evasion of immune surveillance, promigratory cytokine secretion, anoikis resistance, and stemness in tumour cells (Mowers et al., 2017). Autophagy has also been implicated in resistance to chemotherapeutic agents that target rapidly dividing cells, because it promotes tumour cell dormancy (Table 1) (O’Donovan et al., 2011). Accordingly, silencing of autophagic proteins can increase the efficacy of chemotherapeutic agents (Zhang et al., 2015). Autophagy can also improve survival of circulating tumour cells and establishment of the pre-metastatic niche (Mowers et al., 2017), as well as increase tumour cell survival after metastasis (Pavlides et al., 2012; Rebecca and Amaravadi, 2016). Overall, autophagy plays important roles in the regulation of EMT, immune surveillance, and angiogenesis (Suzuki et al., 2010; Tuloup-Minguez et al., 2013; Alizadeh et al., 2018; Wu et al., 2018; Losier et al., 2019).

**TABLE 1:** The tumour promoting properties of autophagy.

The tumour promoting properties of autophagy	—
Primary tumour	Secondary tumour
Increased EMT	Tumour cell dormancy
Increased Motility	Drug resistance
Anoikis resistance	Survival
Immunosuppression	Establishing metastatic
Drug resistance	colonies
Secretes tumour	—
promoting cytokines	—
Cell adhesion turnover	—

Epithelial-mesenchymal transition (EMT).

### Mechanism of TGFβ-induced autophagy

**FIGURE 10:**
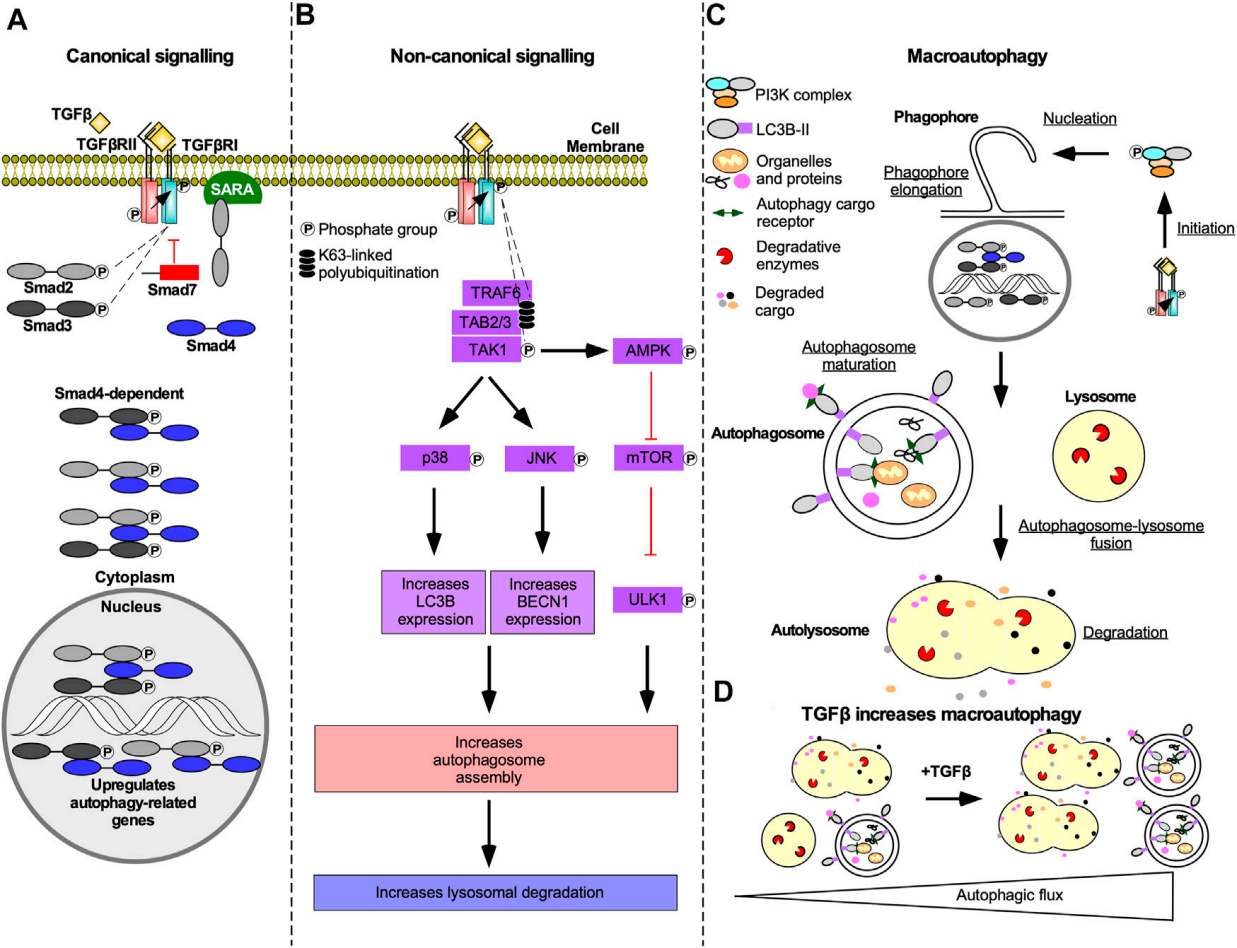
The mechanism of TGFβ-dependent autophagy. **(A)** In Smad-dependent transforming growth factor-β (TGFβ) signalling described in Figure 2, phosphorylated receptor Smads (R-Smads) enter the nucleus with Smad4 and upregulate genes essential to autophagy. Although R-Smad transcription factors may function independently of Smad4, Smad4 knockdown blocked TGFβ-dependent autophagy. **(B)** In Smad-independent TGFβ signalling described in Figure 4, polyubiquitination of tumour necrosis factor receptor-associated factor 6 (TRAF6) recruits TGFβ-activated kinase 1 (TAK1) binding proteins two and 3 (TAB2/3), which leads to TAK1 phosphorylation. Phosphorylated TAK1 activates p38 mitogen-activated protein kinase (p38) and c-Jun amino-terminal kinase (JNK) that phosphorylate several transcription factors that upregulate microtubule-associated protein light chain 3B (LC3B) and beclin1 (BECN1) expression, respectively. TAK1 also phosphorylates 5’ adenosine monophosphate-activated protein kinase (AMPK), which is an inhibitor of an autophagy suppressor called mechanistic target of rapamycin (mTOR). mTOR suppresses autophagy by adding an inhibitory phosphate to uncoordinated-51-like autophagy activating protein kinase 1 (ULK1). LC3B, BECN1, and ULK1 promote autophagosome assembly, which may increase lysosomal-dependent degradation. **(C)** Both Smad-dependent and -independent TGFβ signalling induces macroautophagy. Macroautophagy is initiated when complexes containing ULK1 phosphorylate beclin1. Beclin1 is then primed to form protein complexes that are recruited to the rough endoplasmic reticulum membrane to nucleate phagophores. As the phagophore membranes are elongated with lipids and LC3B, cargo proteins, and organelles, such as mitochondria, are sequestered within autophagosomes. Once phagophore assembly is complete, it forms a mature double membrane vesicle called an autophagosomes. Autophagosomes fuse with lysosomes to generate autolysosomes. The autophagosomes and cargo are degraded by lysosomal proteases. **(D)** Schematic illustrating that in the absence of TGFβ there are few autophagosomes and autolysosomes. In the presence of TGFβ, the number of autophagosomes and autolysosomes is increased.

Both Smad-dependent and -independent TGFβ signalling can contribute to increases in the rate of autophagy (i.e. autophagic flux). Smad-dependent signalling activates transcription of genes essential to autophagy, such as *autophagy-related gene* (*ATG*)*5, ATG7, BECLIN1*, and *DAPK1* (Figure 10A) (Suzuki et al., 2010; Ma et al., 2017). TGFβ can also increase steady-state levels of beclin1, autophagy-related protein (Atg)7, Atg5, uncoordinated 51-like autophagy activating kinase 1 (ULK1), and microtubule-associated protein light chain 3-II (LC3-II) (Xu et al., 2012; Trelford and Guglielmo, 2020). Non-canonical TAK1-mediated TGFβ signalling has also been implicated in regulation of autophagy. Specifically, TGFβ induces phosphorylation and activation of 5’ adenosine monophosphate-activated protein kinase (AMPK) by TAK1 (Herrero-Martín et al., 2009), thereby increasing autophagy as AMPK activates ULK1 and suppresses mTOR (Mcalpine et al., 2013). mTOR antagonizes autophagy through the addition of an inhibitory phosphate to ULK1, which prevents the formation of the autophagy initiating ULK1 complex (Makhov et al., 2014). TAK1 and JNK signalling have also been linked to increased steady-state levels of LC3 and beclin1. LC3 and beclin1 steady-state levels are correlated to the number of autophagosomes, double membrane vesicles that sequester cellular cargo prior to fusing with lysosomes, and increased lysosomal degradation (Figure 10B) (Shin et al., 2013). In support of this, TGFβ increases autophagosomes production, LC3 co-localization with autophagosomes or lysosomes, and autophagosome-lysosome fusion in a variety of cell types (Figure 10C&D) (Alizadeh et al., 2018; Trelford and Guglielmo, 2020).

In non-small cell lung cancer cells transfected with a pMRX-IP-green fluorescent protein (GFP)-LC3-red fluorescent protein (RFP)-LC3ΔGly construct, TGFβ decreased the GFP/RFP ratio, which verified that TGFβ upregulated autophagic flux (Trelford and Guglielmo, 2020). However, the TGFβ-dependent increase in autophagic flux was attenuated by Smad4 knockdown or TAK1/TRAF6/p38 MAPK pathway disruption (Trelford and Di Guglielmo, 2021). In the same cell line system, TGFβ increased the proportion of phosphorylated ULK1 mediated by AMPK and further investigation showed that ULK1 inhibition blocked TGFβ-dependent autophagy (Trelford and Di Guglielmo, 2021; Trelford and Guglielmo, 2021). In summary, Smad-dependent and -independent TGFβ signalling activate autophagy in a ULK1-dependent manner (Trelford and Di Guglielmo, 2021).

### The activation of autophagy through TGFβ augments tumourigenesis

**FIGURE 11:**
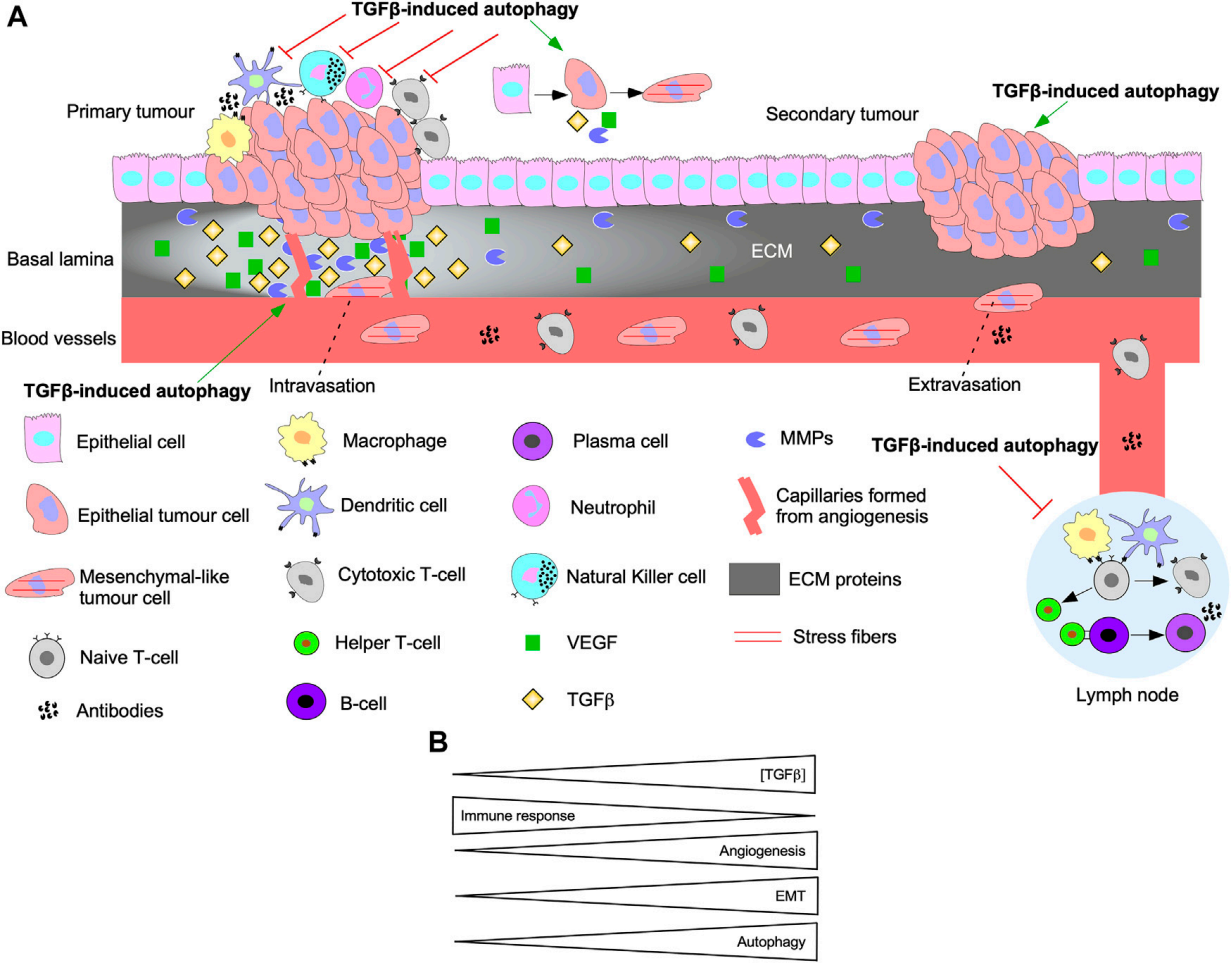
The interplay between autophagy and TGFβ signalling in tumourigenesis. **(A)** A schematic summarizing the effect of TGFβ-induced autophagy on EMT, immune surveillance, angiogenesis, and metastasis. Epithelial cells acquire mutations to the TGFβ pathway until they become cancerous and proliferate rapidly to form the primary tumour. TGFβ-induced autophagy protects tumour cells from the innate immune system (macrophages, dendritic cells, neutrophils, Natural Killer cells) and cells of the adaptive immune system (Naive T-cell, Cytotoxic T-cells, Helper T-cells, B-cells, and plasma cells). Furthermore, TGFβ and autophagy can prevent activation of immune cells that reside in lymph nodes. TGFβ-induced autophagy promotes the release of vascular endothelial growth factor (VEGF) that stimulate angiogenesis. Over time, cells acquire a mesenchymal-like phenotype and release matrix metalloproteinases (MMPs) to breakdown the basal lamina and intravasate into the bloodstream. TGFβ-induced autophagy promotes intravasation because it protects cells that detach from the basal lamina against anoikis-dependent cell death. The mesenchymal-like tumour cells extravasate from the blood vessel at a distant site from the primary tumour. Autophagy is critical for promoting phenotypes to help tumour cells adapt to new environments and establish secondary tumour sites. **(B)** As the concentration of transforming growth factor-β (TGFβ) increases, the immune response is inhibited, whereas angiogenesis, epithelial-mesenchymal transition (EMT), and autophagy are activated.

Autophagy and TGFβ signalling are reciprocally regulated. In fact, autophagy inhibition blocks Smad-dependent TGFβ signalling by impairing TGFβ receptor endocytosis (Trelford and Di Guglielmo, 2022). Also, siRNA targeting of *ATGs* disrupt TGFβ-induced apoptosis and cell cycle arrest (Irimie et al., 2015). TGFβ-induced autophagy has been implicated in EMT, angiogenesis, and immune suppression (Figure 11A). For instance, TGFβ signalling pathways that activate autophagy regulate pro-tumourigenic TGFβ outcomes. Indeed, disrupting Smad4 and TAK1/TRAF6/p38 MAPK signalling pathways blocked TGFβ-dependent E-Cadherin to N-Cadherin shift and stress fiber formation (Trelford and Di Guglielmo, 2022). Attenuation of TGFβ-induced migration has also been reported following inhibition of autophagy (Alizadeh et al., 2018). In pancreatic ductal adenocarcinoma cells, autophagy is required for TGFβ-induced migration, proliferation, and invasion (He et al., 2019; Li et al., 2021). TGFβ-induced autophagy also decreases the expression of proinflammatory cytokines in macrophages (Pokharel et al., 2016). Furthermore, genomic analysis of colon cancer revealed that autophagy upregulates immune checkpoint molecules that dampen the immune response, whereas EMT, TGFβ, and angiogenic pathways were enhanced (Zhu et al., 2020). *In vivo* xenograft models of breast cancer demonstrate that TGFβ-induced autophagy protected fibroblasts from cell death-mediated by nutrient starvation and increased CAF phenotypes (Liu et al., 2016). Although the research of the effect of TGFβ-induced autophagy in tumourigenesis is scarce, data shows that as TGFβ signalling and autophagy are upregulated, angiogenesis and EMT increase whereas the immune response is dampened (Figure 11B) (Bustos et al., 2020).

### Autophagy cargo receptors bridge autophagy and TGFβ signalling

Although there are several catabolic processes that regulate protein quality control in mammalian cells, the UPP and autophagy/lysosome pathway are the two central processes (Wojcik, 2013). Due to difference in substrate selectivity, preparation for degradation, and degradative organelles, the UPP and autophagy do not necessarily compete with one another. Instead, their relationship may be described as compensatory. For instance, when autophagy or the UPP are disrupted, the other major route of protein degradation increases protein turnover to compensate for the disruption (Wojcik, 2013). One explanation is that both lysosome and proteosome-dependent degradation rely on ubiquitination to identify proteins destined for degradation (Lecker et al., 2006; Pankiv et al., 2007; Kirkin et al., 2009). Also, both autophagy and the UPP depend on cargo adaptor proteins such as protein 62/sequestosome 1 (p62/SQSTM1) to deliver substrate proteins (Cohen-Kaplan et al., 2016). Currently, the mechanism of how p62/SQSTM1 decides which pathway receives the ubiquitinated protein remains unknown. Thus far, what has been shown is that p62/SQSTM1 is an autophagy cargo receptor protein that functions in autophagic degradation, regulates EMT, binds to ubiquitin, and is important for TGFβ signalling (Puissant et al., 2012a; Moscat and Diaz-Meco, 2012; Bitto et al., 2014).

**FIGURE 12:**
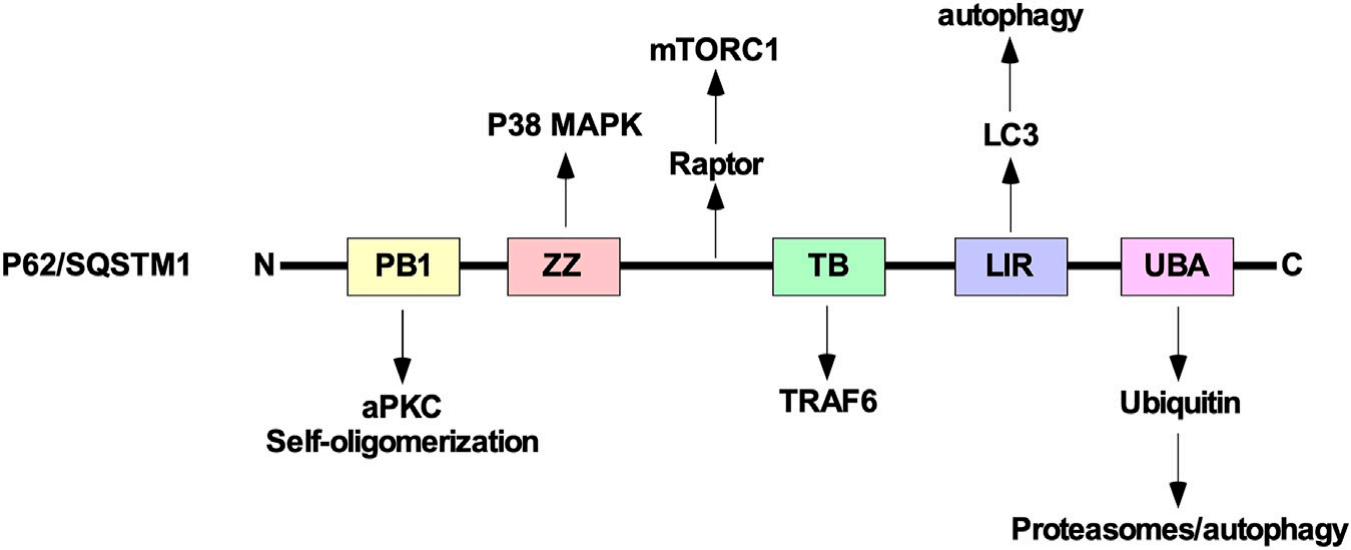
The structure of p62/SQSTM1. From the amino (N)-terminal to carboxyl (C)-terminal, p62/SQSTM1 is comprised of the phox bem1 (PB1), ZZ-type zinc finger (ZZ), tumour necrosis factor receptor-associated factor (TRAF) binding (TB), microtubule-associated protein light chain 3 (LC3)-interacting region (LIR), and ubiquitin-associated (UBA) domains. The PB1 domain allows protein 62/sequestosome 1 (p62/SQSTM1) to interact with atypical protein kinase C (aPKC) and self-oligomerize. The ZZ and TB domain have been shown to interact with downstream transforming growth factor-β (TGFβ) signalling molecules, such as p38 mitogen-activated protein kinase (MAPK) and TRAF6, respectively. Between the ZZ and TB domains, p62/SQSTM1 associates with Raptor, which is a component of mechanistic target of rapamycin complex 1 (mTORC1). The LIR binds to LC3 and is necessary to facilitate selective autophagy. The UBA domain recognizes ubiquitin prior to delivering ubiquitin-conjugated proteins to proteasomes or lysosomes.

P62/SQSTM1 is composed of several domains including a phox bem1 (PB1) domain, ZZ-type zinc finger (ZZ) domain, TRAF binding (TB) domain, LC3-interacting region (LIR), and ubiquitin-associated (UBA) domain. The UBA domain allows p62/SQSTM1 to functions as a ubiquitin receptor protein that targets ubiquitinated proteins to proteasomes (Puissant et al., 2012b; Cohen-Kaplan et al., 2016). In addition to regulating autophagy and the proteasome, p62/SQSTM1 can sequester several downstream TGFβ signalling molecules, including p38 MAPK, TRAF6, and aPKC using the ZZ, TB, and PB1 domains, respectively. These proteins have been implicated in modulating autophagy induction and TGFβ receptor trafficking (Sanz et al., 1999). Furthermore, using the PB1 domain, p62/SQSTM1 self-oligomerizes to sequester intracellular cargo during cell stress or disruption to protein turnover pathways (Lippai and Low, 2014). Also, between the ZZ and TB domains, there is a region of p62/SQSTM1 that interacts with Raptor, a component of mechanistic target of rapamycin complex 1, which is an additional link between p62/SQSTM1 and autophagy (Figure 12).

An image based genome wide small interfering RNA screen in mammalian cells identified Smurf1 as a mediator of selective autophagy (Orvedahl et al., 2011). Since we know that Smurf1 also mediates the UPP, this suggests that TGFβ-specific signalling modulators also have the potential to regulate protein degradation pathways. Therefore, there is evidence of crosstalk between TGFβ signal transduction pathways, autophagy, and the UPP. Given that autophagy, proteasomes, and p62/SQSTM1 regulate TGFβ-dependent EMT (Bertrand et al., 2015; Moon et al., 2017; Alizadeh et al., 2018) and are altered by TGFβ treatment (Bonni et al., 2001; Liang et al., 2020), proteins such as p62/SQSTM1 may be important to understanding the crosstalk between protein degradation pathways and TGFβ signalling. Although the role of p62/SQSTM1 in tumourigenesis is context dependent, it may be an important pharmacological target for regulating TGFβ signalling transduction in cancer (Yuan et al., 2013).

## Targeting TGFβ signalling in cancer therapy

Due to the abnormal TGFβ signalling in tumour cells and elevated TGFβ ligand concentrations in tumour microenvironments, modern adjuvant therapies aim to antagonize TGFβ signalling (Yingling et al., 2004). Although TGFβ antagonists are ineffective at treating tumourigenesis as monotherapies, antagonizing TGFβ as part of combination therapies is promising (Teixeira et al., 2020). Current strategies employed to mitigate pro-tumourigenic TGFβ signalling have been extensively reviewed elsewhere (Sheen et al., 2013; Kim et al., 2021). As such, this review will summarize therapeutic strategies undergoing clinical investigations.

**FIGURE 13:**
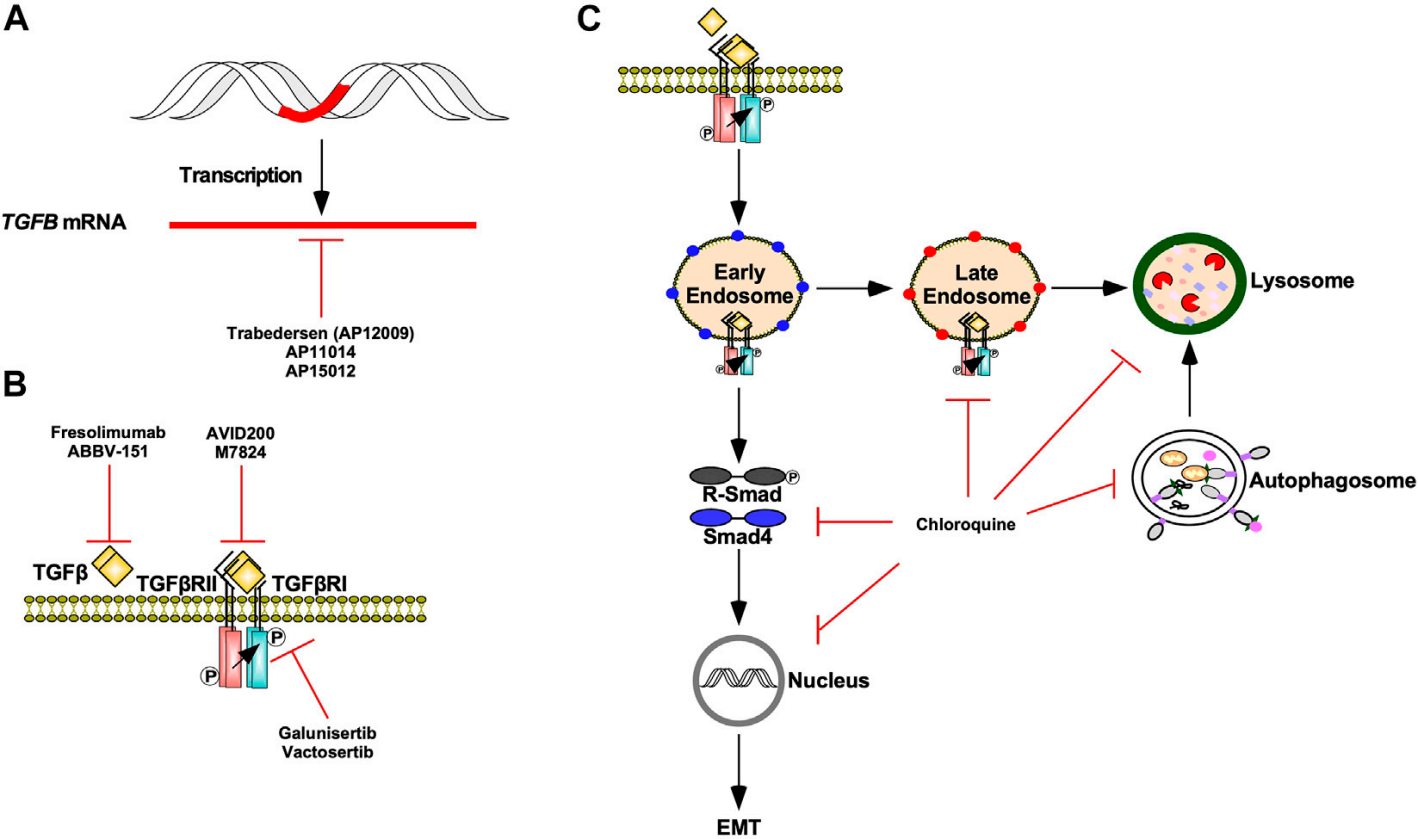
TGFβ signalling targeted therapies. **(A)** Trabedersen (AP12009), AP11014, and AP15012 are antisense oligodeoxynucleotides that decrease *TGFB* expression *via* mRNA targeting. **(B)** Fresolimumab and ABBV-151 are monoclonal antibodies against TGFβ ligands that block TGFβ from binding to TGFβ receptor type II (TGFβRII). AVID200 and M7824 are ligand traps that compete with TGFβRII for TGFβ ligands. Galunisertib and Vactosertib are TGFβ receptor type I (TGFβRI) kinase antagonists. **(C)** Chloroquine is an autophagy inhibitor that blocks autophagosomes and endosomes from fusing with lysosomes as well as lysosomal-dependent degradation. Chloroquine impedes TGFβ receptor internalization and trafficking through early endosome, late endosome, and lysosome membrane compartments. Chloroquine also decreases receptor regulated Smad (R-Smad) phosphorylation, R-Smad nuclear translocation, and TGFβ-dependent epithelial-mesenchymal transition (EMT).

Modern adjuvant therapies antagonize pro-tumourigenic TGFβ signalling by targeting TGFβ ligand production, TGFβ-TGFβ receptor interactions, and TGFβ receptor kinase activity (Kim et al., 2021). Antisense oligodeoxynucleotides, such as Trabedersen (AP12009), AP11014, and AP15012 attenuate the mRNA expression of *TGFβ2*, *TGFβ1*, and *TGFβ1*, respectively. Although AP11014 and AP15012 are in pre-clinical development (Sheen et al., 2013), Trabedersen has proven to be safe and effective and is undergoing phase III clinical trials (Bogdahn et al., 2011). TGFβ-TGFβ receptor interactions are pharmacologically blocked using ligand traps or neutralizing antibodies against TGFβ ligands or TGFβ receptors. AVID200, a TGFβ trap comprised of TGFβRII ectodomains fused to human fragment crystallizable domains, has demonstrated high affinity for TGFβ1 and TGFβ3 in clinical trials (Yap et al., 2020). Furthermore, the success of pre-clinical studies of soluble TGFβRII and betaglycan receptors verify that ligand trapping is an effective approach at antagonizing TGFβ signalling *in vivo* (Bandyopadhyay et al., 2002). As for neutralizing antibodies, Fresolimumab, a pan TGFβ human monoclonal antibody, is in clinical trials for malignant melanoma (Morris et al., 2014). TGFβRI kinase inhibitors, such as Vactosertib and Galunisertib, are safe and effective antagonists of TGFβ signalling and clinical trials assessing their potential in combination therapies are in progress (Figure 13) (Herbertz et al., 2015; Song et al., 2019).

Given that TGFβ protects tumour cells from the immune system and cancer cells stimulate immune checkpoint inhibitory receptors, anti-tumourigenic immunotherapies are being developed to stimulate immune-mediated destruction of tumour cells (Bai et al., 2019). As such, numerous clinical trials are assessing the efficacy of combining immune checkpoint inhibitors alongside TGFβ signalling antagonists (Maruyama et al., 2022). For instance, ABBV-151 and Budigalimab (formerly known as ABBV-181), anti-TGFβ1 and anti-programmed cell death receptor one antibodies, respectively, have begun phase I clinical trials for advanced solid tumours (Powderly et al., 2020). Likewise, the safety and efficacy of Vactosertib or Galunisertib in conjunction with Durvalumab, a monoclonal programed cell death ligand 1 (PD-L1) antibody, are under investigation in lung, pancreatic, colorectal, and gastric cancer clinical trials (Bai et al., 2019). Finally, M7824, a bifunctional fusion protein containing an extracellular TGFβRII domain and antibody against PD-L1, localizes to tumour microenvironments, sequesters TGFβ ligands, and stimulates T-cell immune activity (Figure 13) (Knudson et al., 2018; Paz-Ares et al., 2018; Lind et al., 2020).

Although the dual blockage of immune checkpoint inhibitors and TGFβ signalling is promising, several obstacles with respect to antagonizing TGFβ signalling in tumourigenesis remain. For instance, targeting TGFβ signalling has been successful *in vitro* and in pre-clinical studies; however, these outcomes fail to translate in clinical trials (Teixeira et al., 2020). Limited understanding of the interplay between the numerous proteins involved in TGFβ synthesis, activation, signalling, and signalling crosstalk are among the shortcoming of utilizing modern TGFβ inhibitors in adjuvant combination therapies (Kim et al., 2021). Indeed, the combination of the ubiquitous expression of TGFβ ligands, lack of dosing regimens, and its dual role in tumourigenesis pose a challenge to utilizing TGFβ antagonists in cancer therapy (Sheen et al., 2013).

To date, few autophagy inhibitors have been approved for clinical trials for anticancer therapy. Among those approved, diprotic weak bases, such as chloroquine and hydroxychloroquine, and the proton pump inhibitor, pantoprazole, antagonize autophagy by limiting endosomal and/or lysosomal acidification, which blunts lysosomal fusion and lysosomal hydrolase activity (Beil et al., 1992; Halcrow et al., 2021). However, anti-tumourigenic properties of chloroquine, hydroxychloroquine, and pantoprazole rely on both autophagy inhibition and decreasing glycolysis, lactate production, and cytosolic pH (Halcrow et al., 2021). Despite there being no clinical trials investigating autophagy inhibitors in combination with TGFβ signalling antagonist, *in vitro* studies suggest that chloroquine can disrupt TGFβ signalling (Wu et al., 2018). In Mv1Lu cells, chloroquine antagonized TGFβRII internalization and decreased co-localization with EEA1, Rab7, and LAMP1-positive membrane compartments. Furthermore, R-Smad phosphorylation, R-Smad nuclear translocation, and mesenchymal phenotypes in NSCLC cells treated with TGFβ1 were suppressed by chloroquine (Figure 13) (Trelford and Di Guglielmo, 2022). As such, autophagy inhibitors may be applicable in targeting tumourigenesis driven by aberrant TGFβ signalling without the need to utilize a direct inhibitor of the TGFβ pathway.

## Concluding remarks

This review highlights TGFβ signalling pathways that contribute to homeostasis and tumour biology. TGFβ enhances tumourigenesis by promoting proliferation, immune suppression, angiogenesis, lymphangiogenesis, EMT, EndMT, and autophagy. Components of the TGFβ pathway pharmaceutically targeted in clinical trials are limited to TGFβ synthesis, TGFβ-TGFβ receptor interactions, and TGFβRI kinase activity. Although some combination therapies may improve patient prognosis, the efficacy of TGFβ signalling antagonists are underwhelming. Based on the existing literature, there is an abundance of studies exploring TGFβ-dependent EMT, angiogenesis, and immune suppression. Even though there is still much to be learned about these processes and how they interact with each other to promote tumourigenesis, studies exploring the impact that TGFβ has on other tumour promoting biological processes are scarce. Indeed, further work is needed to explore the relationship between TGFβ and autophagy as well as other processes involved with protein quality control, which may yield new therapeutic approaches in targeting TGFβ-dependent tumourigenesis.
